# Translocation of gold nanoparticles across the lung epithelial tissue barrier: Combining *in vitro* and *in silico* methods to substitute *in vivo* experiments

**DOI:** 10.1186/s12989-015-0090-8

**Published:** 2015-06-27

**Authors:** Gerald Bachler, Sabrina Losert, Yuki Umehara, Natalie von Goetz, Laura Rodriguez-Lorenzo, Alke Petri-Fink, Barbara Rothen-Rutishauser, Konrad Hungerbuehler

**Affiliations:** ETH Zürich, Institute for Chemical and Bioengineering, 8093 Zürich, Switzerland; University of Fribourg, Adolphe Merkle Institute, 1700 Fribourg, Switzerland; EMPA, Swiss Federal Laboratories for Material Science and Technology, 8600 Dübendorf, Switzerland

**Keywords:** 3R, A549, Air-liquid interface, ALICE, Inhalation exposure, MLE-12, MLE12, Epithelial cell monolayers, PBPK model, PBTK model

## Abstract

**Background:**

The lung epithelial tissue barrier represents the main portal for entry of inhaled nanoparticles (NPs) into the systemic circulation. Thus great efforts are currently being made to determine adverse health effects associated with inhalation of NPs. However, to date very little is known about factors that determine the pulmonary translocation of NPs and their subsequent distribution to secondary organs.

**Methods:**

A novel two-step approach to assess the biokinetics of inhaled NPs is presented. In a first step, alveolar epithelial cellular monolayers (CMLs) at the air-liquid interface (ALI) were exposed to aerosolized NPs to determine their translocation kinetics across the epithelial tissue barrier. Then, in a second step, the distribution to secondary organs was predicted with a physiologically based pharmacokinetic (PBPK) model. Monodisperse, spherical, well-characterized, negatively charged gold nanoparticles (AuNP) were used as model NPs. Furthermore, to obtain a comprehensive picture of the translocation kinetics in different species, human (A549) and mouse (MLE-12) alveolar epithelial CMLs were exposed to ionic gold and to various doses (*i.e.,* 25, 50, 100, 150, 200 ng/cm^2^) and sizes (*i.e.,* 2, 7, 18, 46, 80 nm) of AuNP, and incubated post-exposure for different time periods (*i.e.,* 0, 2, 8, 24, 48, 72 h).

**Results:**

The translocation kinetics of the AuNP across A549 and MLE-12 CMLs was similar. The translocated fraction was (1) inversely proportional to the particle size, and (2) independent of the applied dose (up to 100 ng/cm^2^). Furthermore, supplementing the A549 CML with two immune cells, *i.e.,* macrophages and dendritic cells, did not significantly change the amount of translocated AuNP. Comparison of the measured translocation kinetics and modeled biodistribution with *in vivo* data from literature showed that the combination of *in vitro* and *in silico* methods can accurately predict the *in vivo* biokinetics of inhaled/instilled AuNP.

**Conclusion:**

Our approach to combine *in vitro* and *in silico* methods for assessing the pulmonary translocation and biodistribution of NPs has the potential to replace short-term animal studies which aim to assess the pulmonary absorption and biodistribution of NPs, and to serve as a screening tool to identify NPs of special concern.

**Electronic supplementary material:**

The online version of this article (doi:10.1186/s12989-015-0090-8) contains supplementary material, which is available to authorized users.

## Background

Nanomaterials are increasingly used in industrial and consumer products [1–3], so that potentially workers and consumers are exposed to nanomaterials from various sources and via different routes. At the workplace, inhalation is considered to be the primary route of exposure [4]. Consumers can also be exposed to nanomaterials via inhalation through the use of spray products [5–7]. After inhalation, the nanomaterials can deposit on the lung surface and be displaced into the aqueous lining layer, where they may then interact with epithelial cells [8, 9]. These cells form one of the first cellular lines of defense against inhaled nanomaterials. In the human body, the most permeable epithelial barrier is located in the deep lung, lining the alveoli [10]. This barrier, called the air-blood barrier, is only 0.1 to 0.2 μm thick and consists of alveolar epithelial cells type I and surfactant-producing alveolar epithelial cells type II (AT II) [10]. Due to the physiology of the respiratory tract, the alveoli are also the primary region where inhaled particles between 10 and 100 nm can deposit [11]. Thus, great efforts have been devoted to determine adverse health effects in the lungs caused by the inhalation of nanoparticles (NPs), by both *in vitro* and *in vivo* approaches [10, 12–18]. However, little is known about the systemic effects of inhaled NPs, considering that the alveoli have the most permeable epithelial barrier of all uptake routes, and, inhaled NPs have been shown to reach the systemic circulation [19–26]. Furthermore, the factors that enable and determine the pulmonary translocation and biodistribution of NPs are presently largely unknown.

It has been shown that the size of the deposited particles has a large influence on the translocation of NPs through the air-blood barrier. In rats, the translocation is increasing with decreasing particle diameter for both gold [20, 21] and iridium [23, 24] NPs. Another factor that influences the biokinetic behavior is the surface modification of the respective NPs. However, regarding the direction of this influence the literature is inconsistent: While *in vivo* experiments with rats showed a higher translocation for NPs with anionic surfaces as compared to cationic surfaces [19, 20], the opposite was found in *in vitro* experiments using primary alveolar cells from mice [27] and rats [28, 29]. Furthermore, the composition of the pulmonary surfactant layer also seems to influence the translocation kinetics of NPs through the air-blood barrier: After instillation of 10 μg surfactant protein D in mice, the translocation of 22 nm gold nanoparticles (AuNP) decreased approximately by a factor of three after two hours of inhalation exposure [30]. Although this decrease was not significantly different from the control group, the results indicate that the interaction of NPs with proteins in the pulmonary surfactant layer is an important factor.

However, considering the large variety of NPs with different physicochemical characteristics [4], such as chemical composition, particle size and shape, surface modification, aggregation/agglomeration state and specific surface area, the available studies can only be considered as very first steps towards understanding the biokinetic fate of inhaled NPs at the lung epithelial tissue barrier. Furthermore, apart from ethical considerations, considerable technical and financial effort would be necessary to investigate every single type of NP *in vivo* [31], hence highlighting the necessity for alternative methods to determine the toxicokinetics of inhaled NPs.

**Fig. 1:**
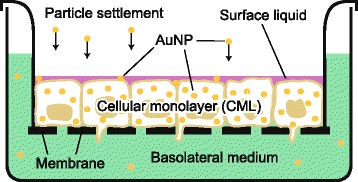
Transwell chamber system that was used to culture and expose the A549 and MLE-12 CMLs. The displayed graphic shows the CMLs at the ALI (24 h after the apical medium was removed and the surface liquid was produced by the cells) and during/shortly after exposure using the ALICE, when the particles settle down and distribute between the individual compartments (surface liquid, cellular monolayer and basolateral medium). Insert surface area: 0.9 cm^2^. Membrane pore size: 3 μm

**Fig. 2:**
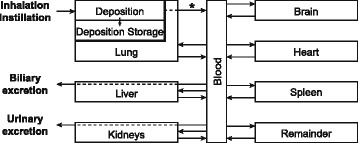
Schematic diagram of the AuNP PBPK model. Dashed lines symbolize the translocation of AuNP through the kidneys and liver to the urine and feces, respectively, and the translocation of AuNP across the lung epithelial tissue barrier (*). Adapted from [39]

In this study, we present a novel two-step approach to assess the biokinetics of inhaled NPs without the use of animal experiments, by combining *in vitro* with *in silico* methods. To this end, first, the translocation kinetics of AuNP across the lung epithelial tissue barrier was determined with alveolar epithelial cellular monolayers (CMLs) at the air-liquid interface (ALI) (Fig. 1). Then, in a second step, their biodistribution to secondary organs was predicted with a physiologically based pharmacokinetic (PBPK) model (Fig. 2). The predictive capability of this approach was evaluated by comparison to available *in vivo* data from the literature [20, 30]. Furthermore, in order to investigate different parameters that influence the pulmonary absorption kinetics, the translocation of AuNP was determined at various time points post-exposure (*i.e.,* 0, 2, 8, 24, 48, 72 h), for different exposure doses (*i.e.,* 25, 50, 100, 150, 200 ng/cm^2^) and for different particle sizes (*i.e.,* 2, 7, 18, 46, 80 nm). For comparison also ionic gold was tested.

The CMLs were exposed to AuNP using the air-liquid interface cell exposure (ALICE) system [32]. With the ALICE system, cells at the ALI can directly be exposed to an NP aerosol, which is an effective way to mimic inhalation exposure [12, 13, 32]. Adenocarcinomic human (A549) and transgenic mouse (MLE-12) epithelial cells were used as CMLs. Both cell types show functional characteristics of AT II epithelial cells, including the development of a surfactant layer [33, 34]. The production of a protein-containing surfactant layer was considered crucial to model the surfactant protein attachment to the surface of the NPs. This surfactant protein-NP interaction may largely influence the biological properties of NPs [35–37] and has been described to be indispensable for *in vitro* studies modeling the lung [38].

To simulate the biodistribution of AuNP we adapted our recently presented PBPK model for titanium dioxide (TiO_2_) NPs [39]. The PBPK model was used in two different ways in this study: (1) to simulate the biodistribution of AuNP that translocate from the lung to the blood and (2) to update the time-dependent pulmonary translocation kinetics measured in animal studies [20, 30], which were compared to our *in vitro* data. The latter was necessary, because in animal studies it is not possible to distinguish mucociliary-cleared from biliary-cleared AuNP in the gastrointestinal tract (GIT) and the feces.

## Results

### AuNP characterization

The physicochemical parameters of the AuNP used are summarized in Table 1 (more details are given in Additional file 1). The particles which were produced cover the whole nano-range (1–100 nm [40]) and are highly stable: the nebulization in the ALICE does not alter the size of the AuNP, and we did not determine a considerable amount of gold below 30 kDa in the basolateral medium 24 h after the exposure in the ALICE, hence the dissolution of particles in the transwell chamber system is insignificant. The relatively high fraction of gold below 30 kDa for the 2 nm particles (8.8 %, Table 1) may be attributed to the particle size distribution of these very small NPs, which includes significant amounts of particles smaller than 30 kDa (30 kDa corresponds to an uncoated AuNP with a size of approximately 1.7 nm. However, the actual cut-off size is probably even lower, due to coating effects in the medium).

**Table 1:** Physicochemical parameters of the AuNP before and after ALICE exposure

	AuNP diameter^a^	2 nm	7 nm	18 nm	46 nm	80 nm
In suspension before ALICE exposure	UV–Vis: Maximum absorption curve^b^ [nm]	No LSPR^h^ band	512	521	529	549
	UV–Vis: Diameter^c^ [nm]	1-2	6-7	16-18	46	80
	DLS: Hydrodynamic diameter [nm]	n.d.^i^	n.d.^i^	20.2	52.4	80.4
	DLS: Polydispersity [%]	n.d.^i^	n.d.^i^	6.2	9.7	13.1
	Surface functionalization	Citrate-THPC^j^	Citrate	Citrate	Citrate	Citrate
	ζ-potential^d^ [mV]	−12.3 ± 0.9	−50.9 ± 1.3	−31.7 ± 1.5	−32.4 ± 1.5	−27.5 ± 2.3
After ALICE exposure	TEM: Particle diameter^e^ [nm]	2.5 ± 1.2^k^	6.5 ± 2.3	19.6 ± 4.2	49.1 ± 10.7	85.5 ± 14.2
	TEM: Circularity^f^ [−]	0.46 ± 0.13	0.60 ± 0.20	0.77 ± 0.13	0.88 ± 0.05	0.86 ± 0.10
	Fraction of gold below 30 kDa in the basolateral medium^g^ [%]	8.80 ± 0.34	0.52 ± 0.16	<LOD^l^	<LOD^l^	<LOD^l^

^a^AuNPs were ordered by size according to the UV–Vis measurements

^b^the complete UV–Vis spectrum can be found in Additional file 1 (Figure S1)

^c^determined as described in Haiss *et al.* [72] (see Methods)

^d^average zeta potential ± standard deviation (SD)

^e^average diameter ± SD as measured by TEM (*n* = 161 to 259). Histograms showing the complete particle size distribution can be found in Additional file 1 (Figure S2)

^f^0 corresponds to an infinitely elongated polygon; 1 corresponds to a perfect circle

^g^determined with an A529 CML 24 h after exposure to 100 ng/cm^2^ AuNP. Mean fraction ± SD (*n* = 3)

^h^LSPR: localized surface plasmon resonance

^i^not detected (size below the LOD of the instrument)

^j^THPC: tetrakis (hydroxymethyl) phosphonium chloride

^k^left tail of the size distribution partly below the limit of detection (<0.9 nm). Hence, mean diameter was probably slightly overestimated; see also Additional file 1

^l^below the limit of detection (LOD depends on size. Approximate LODs are 1.4 % for 18 nm, 1.2 % for 46 nm, 2.3 % for 80 nm)

### CML characterization

**Fig. 3:**
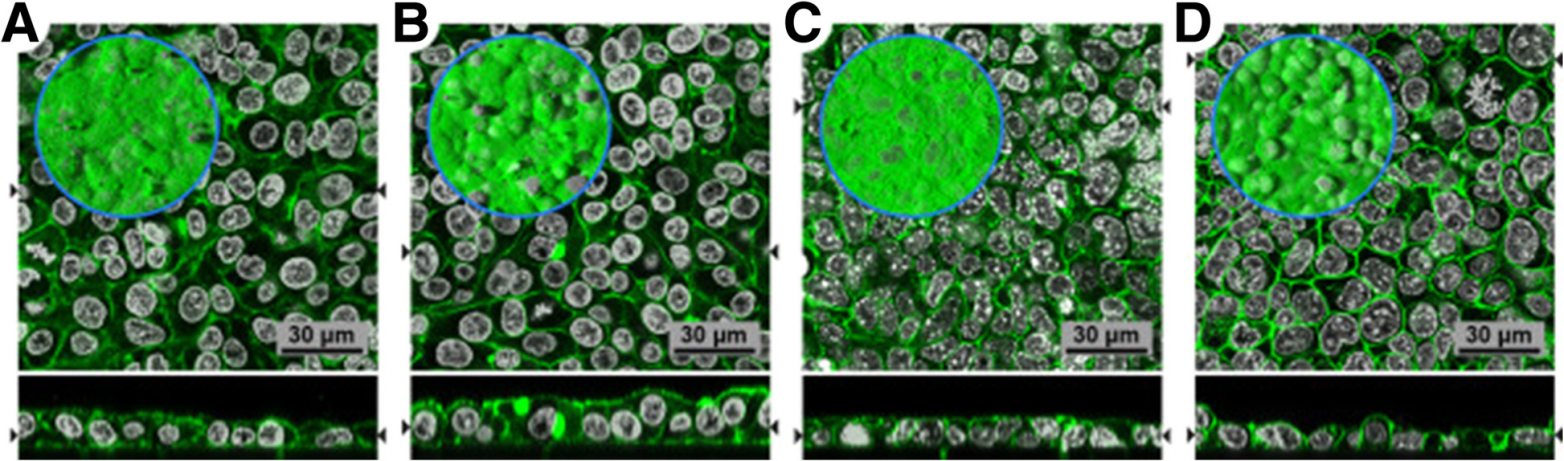
LSM images of cytoskeletal F-actin (green) and the cell nuclei (grey). Orthogonal view of the A549 CML after (**a**) 24 and (**b**) 48 h, and of the MLE-12 CML after (**c**) 24 and (**d**) 48 h at the ALI. (**b, d**) After 24 h at the ALI the CML were exposed to 100 ng/cm^2^ of 18 nm large AuNP. Within the blue circle the top view on the CML is depicted

Laser scanning microscopy (LSM) images of A549 and MLE-12 CMLs after 24 h at the ALI and 24 h post-exposure are depicted in Fig. 3a-d (LSM images 48 and 72 h post-exposure are provided in Additional file 1). In the displayed orthogonal view, the confluent cellular monolayer grown on the membranes can be seen. For both cell types, the CML was well-developed on the entire membrane surface.

The measured surface liquid height on the MLE-12 cells was 68 ± 14 μm (mean ± SD) after 24 h at the ALI. The surface liquid height for the A549 CML was below 10 μm (limit of detection; LOD). Hence, the total surface liquid height on MLE-12 CMLs is approximately four times higher than on A549 CMLs, when the 14 μm liquid film that is formed after nebulization of the AuNP suspension in the ALICE is also considered [32].

**Fig. 4:**
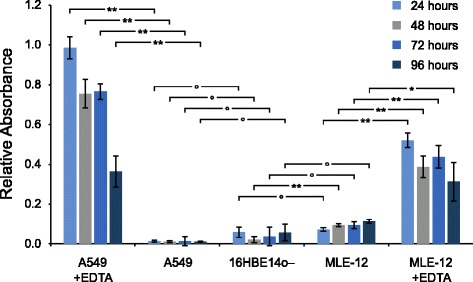
Integrity assessment of A549 and MLE-12 CMLs by Blue Dextran assay. The translocation of Blue Dextran (2000 kDa) through the CMLs after various time points at the ALI. (data are expressed as mean ± SD, n = 3 CMLs; ^┌─┐^ indicates the significance level between two measurements, ° no significant difference, significant difference: * *p* < 0.05, ** *p* < 0.01)

The integrity of the CMLs was evaluated by assessing the permeability to Blue Dextran. The results are depicted in Fig. 4. In brief, 16HBE14o- CMLs were used as reference control due to their known ability to form a tight barrier [41]. As positive control, the CMLs were additionally exposed to ethylenediaminetetraacetic acid (EDTA), which causes the detachment of the CML from the membrane [42] and, hence, increases the translocation of Blue Dextran. Compared to 16HBE14o- CMLs, the permeability of the A549 CML is marginally lower and of MLE-12 CMLs slightly higher at all time points at the ALI (24 to 96 h). However, a significantly increased translocation through MLE-12 CMLs can only be observed after 48 h at the ALI (*p* < 0.01). A detailed discussion on the permeability to Blue Dextran and the time course of the transepithelial electrical resistance (TEER) is provided in Additional file 1.

### Translocation kinetics

**Fig. 5:**
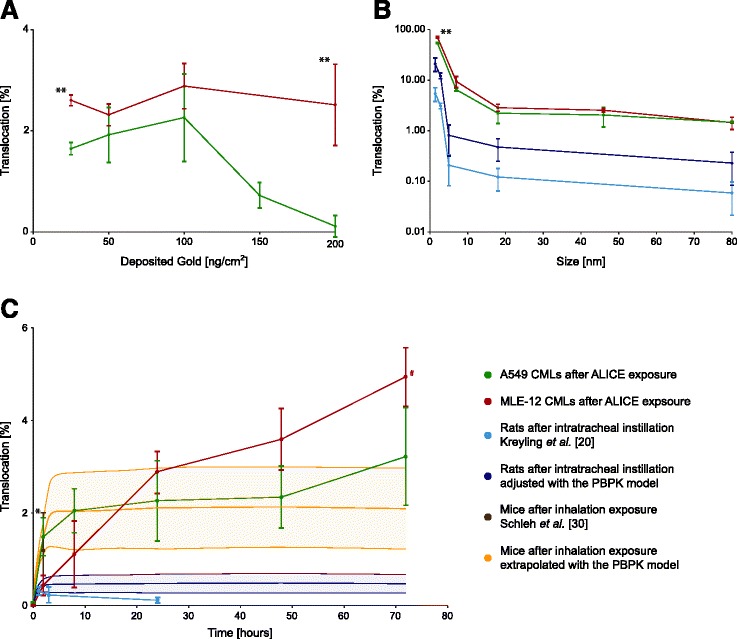
Translocation kinetics of AuNP through A549 and MLE-12 CMLs. (**a**) Translocation fraction of 18 nm AuNP after 24 h for different doses. (**b**) Translocation fraction 24 h after exposure to 100 ng/cm^2^ AuNP for different sizes. (**c**) Translocation fraction of 18 nm AuNP at a dose of 100 ng/cm^2^ after different times post-exposure. The *in vitro* results were compared to *in vivo* data from Kreyling *et al.* [20] and Schleh *et al.* [30]. Kreyling *et al.* determined the translocation fraction of AuNP of various sizes after 24 h (**b**) and of 18 nm AuNP after various time points (c) in female Wistar-Kyoto rats after intratracheal instillation. Schleh *et al.* determined the translocation fraction of 20 nm large AuNP in female C57BL/6 mice following two hours of inhalation exposure (**c**). (detailed information on properties and dose of the AuNP used in the *in vivo* studies may be found in Additional file 1 (Table S1); data are expressed as mean ± SD, *n* = 3 (CMLs, ^#^ *n* = 2), *n* = 4 (rats/mice) and the yellow and blue areas show the uncertainty range using Monte Carlo analysis (PBPK model, *n* = 1000 iterations); significant differences between A549 and MLE-12 CMLs: * *p* < 0.05, ** *p* < 0.01)

The influence of dose, size and incubation time on the translocation kinetics of AuNP was assessed in order to get a comprehensive picture of the translocation kinetics of NPs across the epithelial tissue barrier (Fig. 5). The translocation fraction was defined as the fraction of AuNP that translocated from the surface liquid through the CML to the basolateral medium up to a certain time point post-exposure (Fig. 1).

In Fig. 5a, the translocation kinetics of AuNP through A549 and MLE-12 CMLs are shown for different doses. Both cell types have a similar translocation fraction of around 2 %, which is independent of the applied dose up to 100 ng/cm^2^, however, above this dose the translocation fraction in A549 CMLs decreases significantly. These dose-independent translocation kinetics up to 100 ng/cm^2^ make it possible to compare the translocation fractions of CMLs (obtained at 25–100 ng/cm^2^) to *in vivo* data from literature (obtained at 0.2-8.63 ng/cm^2^ lung surface area, see Additional file 1: Table S1) [20, 30].

In Fig. 5b, the translocation kinetics of AuNP through A549 and MLE-12 CMLs are shown for different particle sizes. Also here, the translocation fractions are almost identical for A549 and MLE-12 CMLs 24 h post-exposure, respectively. A significant difference in the translocation fraction was only observed for 2 nm AuNP, with a significantly higher translocation in murine cells when compared to human cells. The most important outcome, however, is that the size-dependent translocation fraction is inversely proportional to the particle size and, although approximately one order of magnitude higher, changes almost in parallel in the *in vitro* measurements with the *in vivo* measurements by Kreyling *et al.* [20].

**Fig. 6:**
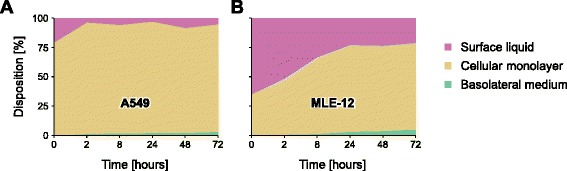
Disposition of 18 nm large AuNP in the transwell chamber system. (**a**) A549 and (**b**) MLE-12 CMLs after various time points at an exposure dose of 100 ng/cm^2^. The results of the translocated fraction (green areas) are presented in more detail in Fig. 5

In Fig. 5c, the translocation kinetics of AuNP through A549 and MLE-12 CMLs are shown for different time points post-exposure. For both cell types, a sharp increase in the translocation fraction can be observed during the first couple of hours, followed by a sharp flattening between 8 and 24 h post-exposure. Between 24 and 72 h post-exposure the amount of translocated AuNP is only increasing slowly. This change in the translocation kinetics 24 h post-exposure may be an indication that the AuNP follow different pathways through the CMLs. The only significant difference between the translocation kinetics of the two cell lines is the much higher translocation fraction that can be observed in A549 CMLs two hours post-exposure. The same sharp rise in A549 CMLs within the first two hours post-exposure can also be seen *in vivo* for the PBPK-adjusted translocation fractions based on Kreyling *et al.* [20] and in the study by Schleh *et al.* [30]. The slower rise in the translocation fraction within the first couple of hours post-exposure in MLE-12 CMLs (*i.e.,* the fraction of AuNP in the basolateral medium) is directly related to the slower clearance of AuNP from the surface liquid (Fig. 6a-b).

**Fig. 7:**
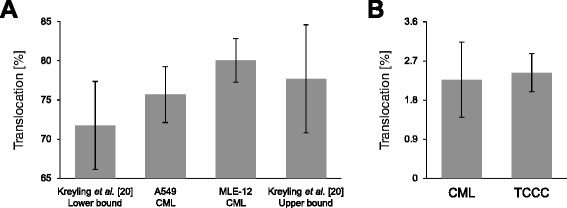
Translocation of (**a**) ionic gold and (**b**) AuNP for two cell models of different complexity. (**a**) Comparison of the translocation fraction of ionic gold after 24 h: CMLs treated with 100 ng/cm^2^ gold were compared to *in vivo* data from Kreyling *et al.* [20], who determined the biodistribution in female Wistar-Kyoto rats after intratracheal instillation (1 μg gold). Two extreme scenarios are presented for the translocation fraction from Kreyling *et al.* For the lower bound, we assumed that all of the gold that was recovered from the GIT and feces had not been translocated, but instead had been cleared via the mucociliary pathway directly from the lungs to the GIT. For the upper bound, we assumed that all of the gold in the GIT and the feces was first taken up and then cleared via the biliary pathway to the GIT. (**b**) Comparison of the translocation fraction of 18 nm large particles in an A549 CML and a TCCC system 24 h after exposure to 100 ng/cm^2^ AuNP. (data are expressed as mean ± SD, *n* = 3 (CML/TCCC) and *n* = 4 (rats))

For comparison, we evaluated the translocation of ionic gold through the CMLs and compared the results to *in vivo* data for ionic gold from Kreyling *et al.* [20] (Fig. 7a). No significant difference in the translocation between any of the *in vitro* experiments and the boundary levels for the *in vivo* studies was observed (Fig. 7a). In all depicted cases, the translocation fraction of ionic gold was around 75 %, which is very close to the translocation fraction that was observed for 2 nm AuNP (A549: 54.2 ± 1.4 %; MLE-12: 71.0 ± 2.8 %; mean ± SD).

Finally, considering that a CML is a very simplified cellular model of the lung epithelial tissue barrier, we also compared the translocation kinetics of 18 nm AuNP through A549 CML with a more sophisticated triple cell co-culture (TCCC) system. In a TCCC system, macrophages and dendritic cells occupying the apical and basal side of the alveolar epithelium, respectively, are also contained in the cellular model [43]. As can be seen in Fig. 7b, there is no significant difference between the translocation of AuNP through the CML and the TCCC system, hence, a CML is sufficient to model the translocation of the investigated AuNP through a cellular representation of the air-blood barrier.

**Fig. 8:**
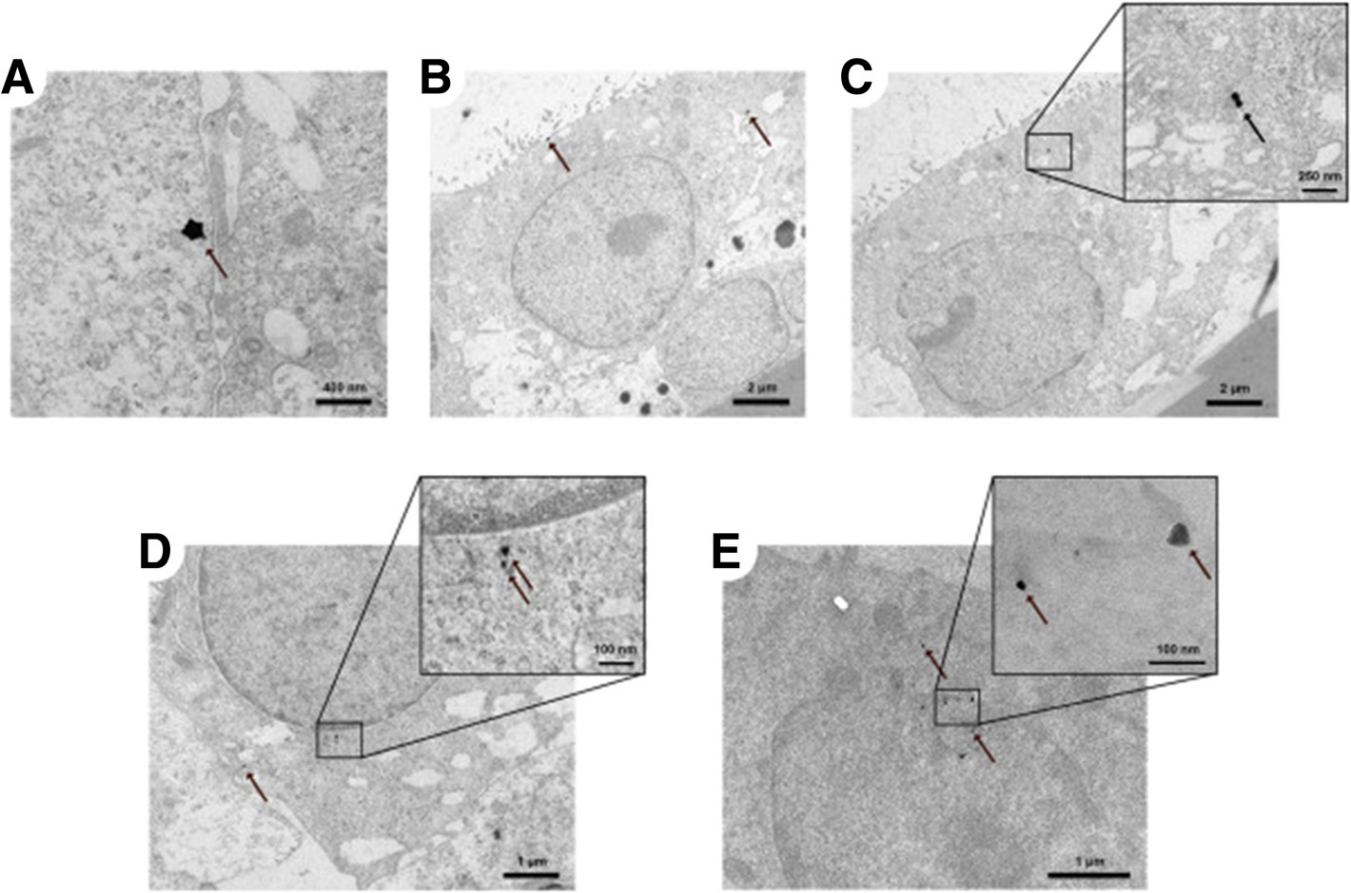
TEM images of intracellular particles in the A549 CML. (**a**) Agglomerates, (**b, c**) small agglomerates and (**d**) single particles in the cytoplasm and (**e**) particles next to the cell nucleus 24 h after exposure to 100 ng/cm^2^ 18 nm AuNP in the ALICE system. In (**b**) and (**c**) the surface of the monolayer (top left) and the PET membrane on which the CML were grown (bottom right) can be seen. The arrows are pointing towards the particles. (e: without lead citrate and uranyl acetate staining)

Transmission electron microscopy (TEM) images of the A549 CML 24 h after exposure to 18 nm AuNP are depicted in Fig. 8a-e. As can be seen, the particles are mainly present as single particles or as small agglomerates, consisting of only a few particles. Larger agglomerates, such as in Fig. 8a, were only observed sporadically. Particularly interesting is that the particles and agglomerates were freely distributed within the cytoplasm (Fig. 8b-d) and are also observed close to the cell nucleus (Fig. 8e). Particles in vesicles were not observed. TEM images for the 2, 7 and 46 nm AuNP can be found in Additional file 1. In brief, the 7 and 46 nm large particles showed the same distribution pattern as the 18 nm AuNP (Additional file 1: Figure S7). The 2 nm AuNP could not be distinguished from the background, even though we prepared samples without lead citrate and uranyl acetate staining. For 2 nm AuNP only one single agglomerate was detected (Additional file 1: Figure S7).

### PBPK modeling

As mentioned above, it was not possible to unambiguously allocate the AuNP recovered in the feces in the animal experiments to the two pathways biliary excretion of AuNP (with translocation) and mucociliary clearance of AuNP (without translocation) [20, 30] that were compared to our CMLs set-up. Hence, *in vivo* the translocation fractions were calculated based solely on the recovered gold from secondary organs, making it difficult to compare the *in vivo* data to the *in vitro* results presented in this paper. One way to overcome this problem is PBPK modeling, which makes it possible to account for the biliary excretion of NPs *in vivo* and adjust accordingly the measured translocation fraction to yield the total translocation of NPs from the lung to the blood circulation.

In Fig. 5c, the adjusted translocation fractions for the data of Kreyling *et al.* [20] are depicted up to 72 h after intratracheal instillation of 18 nm AuNP in rats. The adjusted curve shows a sharp increase in the translocation fraction during the first two hours post-exposure, which is in good agreement with the translocation kinetics in A549 CMLs. Steady state is reached approximately three hours after exposure. In total, the adjusted translocation fraction is 3.9 times higher than the one originally measured *in vivo* 24 h post-exposure. Unfortunately, *in vivo* the translocation was only assessed up to 24 h post-exposure, so it is not possible to confirm the biphasic translocation kinetics observed *in vitro* with *in vivo* data.

In Fig. 5c, the translocation fraction in mice after 2 h of inhalation exposure from Schleh *et al.* [30] is also illustrated. As can be seen, the *in vivo* translocation fraction is in agreement with the translocation fractions measured in A549 and MLE-12 CMLs 2 h post-exposure. With the PBPK model it was further possible to determine the complete time course of the translocation by fitting the translocation rate in the PBPK model to the translocation fraction reported by Schleh *et al.* [30]. The obtained time course is in good agreement with the *in vitro* data of both cell types (Fig. 5c). The measured translocation time course of the A549 CML and the extrapolated translocation time course in mice are practically identical, whereas MLE-12 differs during the first 8 h post-exposure.

**Fig. 9:**
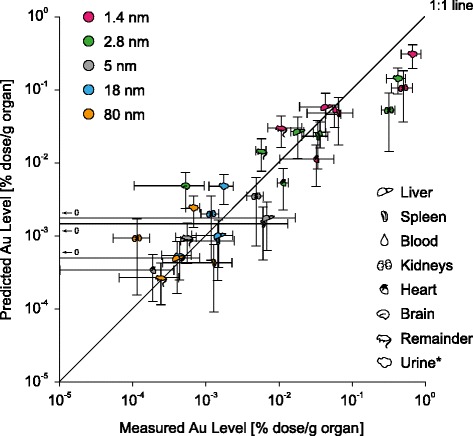
Comparison of the PBPK model to biokinetic data from female Wistar-Kyoto rats [20] 24 h after intratracheal instillation of various sizes of AuNP. (only organs where the gold levels in all rats were above the LOD are depicted; data are expressed as mean ± SD, *n* = 4 (rats) and *n* = 1000 iterations (PBPK model), *unit for urine: % dose)

Finally, the 24 h post-exposure translocation fractions of the 1.4, 2.8, 5 and 80 nm large AuNP were adjusted by the same factor that was determined for the 18 nm large AuNP with the PBPK model (Fig. 5b). These adjusted translocation fractions were further used to fit the translocation rates and to simulate the biodistribution of the AuNP with the PBPK model (Fig. 9; Note that the 18 nm data set cannot be considered independent, because it was already used to add the inhalation/instillation pathway to the PBPK model and adjust the translocation fractions of the other particle sizes). Although the biodistribution is modeled size-independently in the PBPK model, all predicted organ and urine gold levels of all particle sizes fell close to the 1:1 line. Sole exceptions were the kidneys’ and urine gold levels for the 1.4 nm and 2.8 nm AuNP, which were underestimated by the PBPK model.

## Discussion

### AuNP characterization

AuNP were used as model NPs due to their low toxicity [44–46], high stability under biologically relevant conditions [20, 45], high electron density and the availability of good quality *in vivo* studies [20, 30], which were used to evaluate the predictive capability of our *in vitro* and *in silico* results. Furthermore, to minimize the influence of the coating on the translocation kinetics, the AuNP were stabilized with citrate. Citrate is known to be easily replaced by proteins in biological media [47, 48] such as the surface liquid lining the CML and, thus, allows the particles to quickly form a protein corona.

### Choice of cell types

In contrast to primary cells, cell lines are often preferred due to the easy and reproducible use of cell cultures as well as the purity of cell types. MLE-12 and A549 cells were used, which are mouse and human alveolar type II like cell lines [33, 49], respectively, that are well characterized and widely used as *in vitro* lung epithelial cell models. The advantage of those two cell types is the production of surfactant [33, 34], however, their relevance to study NP translocation is often questioned [50], because the expression of tight junction proteins is not as pronounced as in other cell lines or primary cells. The two cell lines used in the current study were grown under controlled culture conditions revealing a confluent and dense monolayer also when cultured at the ALI as shown by the Dextran Blue translocation assay as well as by LSM images of the spatial cell morphology. Especially at the ALI the cells show a denser monolayer compared to cells cultured in suspension [51]. We also did not observe any NPs between cells, but always inside cells. Therefore their use to study the translocation of the here applied AuNPs seems to be justifiable. Other cell lines such as the recently immortalized type II cells that exhibit a type I like phenotype described by Kemp and colleagues [52] or primary cells derived from mice or human lung biopsies could, however, be considered in future studies.

### Translocation kinetics

The determined translocation kinetics of AuNP through A549 and MLE-12 CMLs are in good agreement with kinetic data for AuNP in mice [30] and rats [20] *in vivo.* This is particularly true for the size-dependent translocation kinetics, where the *in vivo* and *in vitro* curves are almost parallel (Fig. 5b). Hence, exposing CMLs to NPs in the ALICE system probably is an adequate model to describe the translocation of NPs through the air-blood barrier after short-term exposure. Further, there are four notable observations that shall be discussed in more detail below, namely, (1) the significantly different translocation fractions between A549 and MLE-12 CMLs two hours after exposure in the ALICE system, (2) the sharp decrease in the translocation efficiency of AuNP through A549 CMLs at doses above 100 ng/cm^2^, (3) the higher translocation fractions determined with the CMLs in the ALICE system as compared to intratracheally instilled AuNP in rats, and (4) the trend that the smaller the AuNP become, the more their translocation kinetics converge to the translocation kinetics of ionic gold.

The first two observations are probably related to the different surface liquid heights between A549 and MLE-12 CMLs. While the surface liquid height observed for A549 CMLs is in good agreement with the height of 4.5 ± 0.7 μm (mean ± SD) that was reported previously for primary human AT II cells [53], the observed surface liquid height for MLE-12 CMLs is approximately four times higher than measurements of Thompson *et al.* [54] for primary rodent AT II cells (15 μm). This unphysiologically high surface liquid height has a threefold influence on the interaction between the NPs and the cells [55, 56]. First, it prolongs the time that the particles need to sediment to the surface of the cells. Second, it decreases the concentration gradient between the surface liquid and the basolateral medium. Third, it affects the likelihood for NPs to form agglomerates. In our experiments, an increased sedimentation time and/or decreased concentration gradient is most likely responsible for the significantly slower clearance of AuNP from the surface liquid and consequently a lower translocation fraction in MLE-12 CMLs as compared to A549 CMLs two hours post-exposure. After the first two hours, the translocation kinetics of both cell types are comparable with each other, implying that it takes 2 h until the AuNP get into contact with the MLE-12 CML due to the unphysiologically high surface liquid height. Furthermore, the pattern for the translocation kinetics of AuNP at different doses seems also to be related to the height of the surface liquid, such that while the translocation fraction in MLE-12 CMLs is the same up to the maximum investigated dose of 200 ng/cm^2^, there is a sharp decrease of the translocation fraction in A549 CMLs above a dose of 100 ng/cm^2^. A reason for this behavior may be the higher particle concentration in the surface liquid of A549 CMLs and hence a higher likelihood of agglomeration at high doses. For example, Brandenberger *et al.* [12] observed a larger amount of agglomerates in A549 cells after exposure to 561 ng/cm^2^ AuNP (15 nm, citrate-coated) in the ALICE system as compared to 61 ng/cm^2^ of the same particles. Also, as shown in our experiments, the translocation efficiency is decreasing with increasing particle diameter, which can be explained by the size of the corona around the NPs, which changes in the presence of biological fluids (e.g., lung liquid lining layer), or by the formation of agglomerates. This observation clearly highlights the importance of experimental *in vitro* set-ups that properly mimic the deposition of NPs in the lung, such as the ALICE system, and illustrates that unphysiological experimental conditions, such as submersed cultures, may bias the translocation kinetics of NPs. In the present study we have observed single 18 nm sized AuNP free in the cytoplasm, which is in agreement with the study from Brandenberger *et al.* [12] who applied a quantitative approach with TEM and found citrate-stabilized AuNP of a similar size in the cytoplasm. However, the cytoplasm was not the preferred compartment and the majority of the particles were found in vesicles.

The third important observation from our *in vitro* experiments is that the translocation of AuNP across the air-blood barrier of rats after intratracheal instillation is overestimated by the CMLs, even though the translocation of AuNP across the air-blood barrier of mice after inhalation exposure is well reflected by the CMLs. In our opinion, this difference is not primarily linked to the species used, *i.e.,* mouse and rat, because our *in vitro* experiments show that the translocation kinetics of AuNP are very similar between human and murine cells, implying that there is no species-specific mechanism for the investigated AuNP. However, there are two factors that have been reported before to influence the distribution of particles in the lung and the pulmonary absorption of these particles. Factor 1: the method of delivery may greatly determine the distribution of particles in the lung. After inhalation, the particles are usually much more uniformly distributed within the lung as compared to intratracheal instillation [57]. Hence, after intratracheal instillation there might be regions in the lung with relatively high particle densities, which may, as described above, increase the likelihood of agglomeration and consequently decrease the translocation of particles across the air-blood barrier. Factor 2: the surface modification of NPs may greatly influence the translocation of deposited NPs across the air-blood barrier [19, 20, 27–29]. In fact, in the *in vitro* and *in vivo* experiments discussed in this work, different surface modifications have been used. Schleh *et al.* [30] used unmodified AuNP (produced by a spark ignition generator), Kreyling *et al.* [20] used sulfonated-triphenylphosphine-modified (1.4, 5, 18 and 80 nm) and thioglycolic-acid-modified (2.8 nm) AuNP and we used citrate-modified AuNP. Although the zeta potential is similar among all types of AuNP, different surface modifications may lead to different types of protein coronas. The protein corona largely determines the biological properties of the NPs [35–37] and, thus, has the potential to directly influence the translocation kinetics. These considerations further explain the good agreement between our *in vitro* results and the data from Schleh *et al.* [30], since citrate can easily be replaced by proteins [47, 48] and, thus, it is not surprising that citrate-modified AuNP behave similar to unmodified AuNP. However, to fully understand the influence of the surface modification of NPs on the protein corona and translocation kinetics across the air-blood barrier more research is necessary.

The fourth, particularly interesting observation is that the smaller the AuNP are, the more their translocation kinetics are similar to the translocation kinetics of ionic gold. The determination of the translocation pathways across the CMLs was not within the focus of this work, but the results suggest that very small NPs follow similar or even the same pathways as ionic gold. So far, several pathways have been described for different types of NPs and different surface modifications, but these findings are not substantial and consistent enough to link particle properties to specific pathways. Yacobi and colleagues [28, 29] used rat alveolar epithelial cell monolayers to investigate the translocation of polystyrene NPs at different sizes (20 and 100 nm) and negative (carboxylate-modified, sulfate-modified or aldehyde-sulfate-modified) and positive (amidine-modified) surface charges. The authors came to the conclusion that the NPs primarily follow transcellular pathways to traffic across the CML and that the translocation takes place via diffusion. On the contrary, for the same types of polystyrene NPs, different pathways have been proposed for mouse alveolar epithelial cell monolayers [27]. While polystyrene NPs with a positive surface charge mainly crossed the CML via transcellular pathways, which involved clathrin- and dynamin-dependent endocytosis, polystyrene NPs with a negative surface charge followed paracellular and non-endocytic transcellular pathways. In addition, for quantum dots (negative surface charges, hydrodynamic diameter 25 nm) it was reported that they use para- and transcellular pathways to cross rat alveolar epithelial cell monolayers [58]. Hence, the data indicates that the translocation does not take place via endocytic pathways (*i.e.,* caveolin-, clathrin- and dynamin-mediated).

However, since all of these studies were carried out with submerged cultures, the results cannot directly be compared to our translocation data obtained at the ALI. Still, a trend towards para- and transcellular diffusion is apparent for negatively charged NPs. This trend is also reflected in three ways in our data. First, the AuNP can mainly be observed freely distributed in the cytoplasm and not in vesicles. Second, the similar translocation kinetics of A549 and MLE-12 CMLs, and the good agreement between the *in vitro* and *in vivo* data indicate that endocytic pathways, which may differ between species, do not play an important role. Third, the translocation fraction was similar in an A549 CML and a TCCC model, which also suggests a minor role of endocytic pathways. Therefore, these results highlight that exposure of CLMs at the ALI to NPs in the ALICE system is a promising fast and cheap method to investigate the translocation pathways of NPs across the lung epithelial tissue barrier in more detail in the future. However, what yet needs to be demonstrated with the presented model is the capability to predict the translocation kinetics of other types of NPs, e.g., those with positive surface charge, and of agglomerates, for which the usage of endocytic pathways across the lung epithelial tissue barrier has been reported before [27, 59]. To enable this, also more of reliable *in vivo* data are needed.

### PBPK modeling

The disposition of AuNP in rats could successfully be predicted with the PBPK model by using the permeability of the different capillary wall types as a basis to model the biodistribution of the NPs. Interestingly, the same distribution and excretion rates as in our recently presented PBPK model for TiO_2_ NPs [39] could be used, which demonstrates that the biokinetics of both particle types are very similar *in vivo*. Solely, the translocation rate from the organs to the blood had to be slightly increased for AuNP as compared to TiO_2_ NPs. The reason for this difference is unknown, but since we could already show that the size and surface modification have a minor influence on the biodistribution of NPs [39, 60], this difference might be simply related to the chemical properties or to the concentration gradient between the organs and the blood. However, since we could already successfully model the biodistribution of AuNP, TiO_2_ NPs [39], silver NPs [60] and silicon dioxide NPs (unpublished data) with the same model structure by simply varying the retention time of the particles in the organs, this parameter may warrant further research.

Several observations that were made by Kreyling *et al.* [20] on the biokinetics of AuNPs had already been suggested by earlier studies with our PBPK model [39, 60] and, thus, further confirm its validity. For instance, after intratracheal instillation of negatively and positively charged 2.8 nm AuNP in rats, the same biodistribution resulted [20]. This behavior was also predicted with the PBPK model. For silver NPs, it was possible to model the biodistribution of polyvinylpyrrolidone (PVP) and carboxymethyl cellulose coated and uncoated particles, without considering the surface conditions in the model [60]. Also, the size-independent biodistribution of AuNP *in vivo* [20] was already observed with the PBPK model for silver NPs [60]. However, the size-independent distribution holds only true for low internal doses of NPs in the PBPK model. At high internal doses, the uptake of NPs by compartments of the mononuclear phagocytic system, located in the liver, lungs and spleen [61], also need to be considered in the model [39, 60]. This increased distribution of NPs to the liver, lungs and spleen seems to be dose- [62] and at high internal doses also size-dependent [63] and can also be seen *in vivo* for AuNP [64]. The reason for the different distribution patterns observed for low and high internal doses remains unknown, but as we have speculated before on the basis of the results of the PBPK model this might be related either to agglomeration or to a substantial alteration of the protein corona due to a deficit of specific proteins at high levels of NPs in the blood [39, 60].

Interestingly, the PBPK model could also be used to model the biodistribution of AuNP that were smaller than 15 nm, even though 15 nm had been previously defined as the lower size limit of the model [60]. This limit was based on the 6 to 15 nm capillary pore sizes of the kidneys [65]. Below this size limit it was hypothesized that NPs can translocate through the pores of the capillary walls of the kidneys [39, 60] and, thus, very small NPs are much easier transported to the kidneys and excreted in urine [64, 66]. This behavior explains also the underestimation of the *in vivo* kidneys’ and urine gold levels [20] of the 1.4 nm and 2.8 nm AuNP by the PBPK model. However, for the 5 nm AuNP this behavior cannot be observed. This discrepancy can most likely be attributed to the formation of a protein corona, which increases the diameter *in vivo*. In fact, the reported critical diameter for an increased excretion of NPs in urine was reported to be around 5 nm [64, 66] and, thus, considerably below 15 nm. Nevertheless, apart from kidneys’ and urine gold levels of 1.4 nm and 2.8 nm AuNP, the gold levels of all other organs could be predicted by the PBPK model after intratracheal instillation of AuNP in rats [20]. This shows that the model is in fact applicable for NPs below 15 nm, but the levels in the kidneys and the urine may be underestimated.

In the study of Kreyling *et al.* [20], the translocation through the air-blood barrier was also investigated for one particle size above the nano-range (200 nm). The maximum particle size that can be described by the model is 150 nm [60], and is based on the pore size of the liver capillaries [65]. Hence, we have not included particles above the nano-range in our experiments. Particles above this size limit may have difficulties passing through the pores of the capillary wall of the liver and, thus, it can be expected that they are hardly excreted via the biliary pathway. In fact, this size threshold for the biliary excretion of particles was already shown before for intravenously injected AuNP in rats [64] and if this assumption is considered in the PBPK model, the organ gold levels of 200 nm large gold particles [20] can be predicted well by the PBPK model (Additional file 1: Figure S11 and S12). Based on these considerations, the comparably high translocation of 200 nm AuNP through the air-blood barrier that was reported by Kreyling *et al.* [20], may simply be related to a decreased excretion efficiency and, thus, a higher retention time for 200 nm large particles in the body of the rat.

## Conclusion

Our work shows how *in vitro* results obtained by CMLs at the ALI can be combined with PBPK modeling to assess the biokinetics of inhaled NPs. The time- and size-dependent translocation of AuNP across the air-blood barrier in animals is in good agreement with the results obtained with the CMLs. The translocation kinetics are well predicted for mice after inhalation exposure, whereas for rats after intratracheal instillation, the translocation is slightly overestimated, which might be related to the method of delivery or to the fact that different surface modifications were used as compared to the *in vitro* experiments. However, the very same variation with size can be observed in the *in vitro* and *in vivo* data. At exposure doses in the range of realistic exposures (e.g., occupational exposure to 3 mg/m^3^ TiO_2_ NPs [67] for a day), a dose-independent translocation of AuNP across the CML can be observed. In addition, it was possible to demonstrate that there is hardly any difference between murine and human AT II epithelial cell lines, indicating that pulmonary absorption does not differ between rodents and humans for the AuNP used (provided that the NPs reach the same regions of the lung). The smaller the NPs were, the more similar to ionic gold was their disposition in the transwell chamber system. This shows that very small NPs can cross the CML as easily as ionic gold and, thus, indicates that they follow similar or even the same pathways across the blood-air barrier.

The biodistribution of AuNP could successfully be described with a PBPK model that was originally developed for TiO_2_ NPs, and which used similar assumptions as a PBPK model for silver NPs. This raises the question of whether the biokinetics of inorganic NPs is generally very similar *in vivo*. Also for AuNP, the biodistribution is governed by the permeability of the different capillary wall types. Several predictions regarding the biodistribution of NPs that were based on the PBPK model are further supported by the data from Kreyling *et al.* [20]. Most importantly: (1) the minor influence of the surface modification on the biodistribution, (2) an insignificant uptake of particles by the phagocytic cells at low internal exposure and (3) the size-independent biodistribution at low internal exposure, except for increased uptake of very small NPs (smaller than the capillary pores of the kidneys) by the kidneys and excretion in the urine.

The most important outcome of this work is that *in vitro* modeling of the pulmonary absorption with the ALICE system in combination with the presented *in silico* modeling of the biodistribution is closely reflecting the *in vivo* behavior of AuNP. Hence, the presented approach has the potential to reduce short-term animal studies, which aim to assess the pulmonary absorption and biodistribution of NPs. Furthermore, the similar translocation kinetics observed in *in vivo* and *in vitro* experiments indicate that AuNP follow the same pathways through the air-blood barrier in animals as observed for the CMLs at the ALI. Hence, the presented setup may be a relatively cheap and fast *in vitro* model to determine the translocation pathways of NPs through the air-blood barrier, which is also suitable to assess the influence of e.g., the chemical composition, particle size and shape, surface modification, aggregation/agglomeration state and specific surface area.

## Methods

### Gold nanoparticles

#### Synthesis

All investigated sizes of AuNP (2, 7, 18, 46, 80 nm) were synthesized in our laboratory.

The 2 nm gold particles were synthesized immediately before nebulizing in the ALICE. We followed the method described by Yong *et al.* [68], but slightly adapted it by the addition of sodium citrate (Fluka, Sigma-Aldrich Chemie GmbH, Buchs, Switzerland). In brief, 0.5 mL of freshly prepared 1 M NaOH (Merck, Merck (Schweiz) AG, Zug, Switzerland) and 1 mL of tetrakis (hydroxymethyl) phosphonium chloride (THPC) solution, obtained by adding 12 μL of 80 % THPC in water (Sigma-Aldrich, Sigma-Aldrich Chemie GmbH, Buchs, Switzerland) to 1 mL of millipore water, were added to a beaker that contained 45 mL of millipore water. The mixture was then magnetically stirred for 20 min at approximately 100 rpm. After the first 5 min of stirring 10 mL of 5 mM chloroauric acid (HAuCl_4_°3H_2_O, from gold (III) chloride trihydrate; Sigma-Aldrich) and after 20 min 400 μL of 40 mM sodium citrate was added. The final concentration was 170 μg/mL for the 2 nm AuNP.

The 7 nm gold particles were prepared as described by Jana *et al.* [69]. In brief, 2 mL of 40 mM sodium citrate solution were mixed with 1.2 mL of 24.2 mM HAuCl_4_ in 90 mL of millipore water. 1 mL of ice-cold 20 mM sodium borohydride (NaBH_4_; Sigma-Aldrich) was added to the stirred solution, which resulted in the formation of a brownish red dispersion. Vigorous stirring of the gold dispersion was continued for 5 min. After stirring, the dispersion was washed by ultra-filtration at 4500 rpm during 1 h, and redispersed in millipore water. The final concentration was 300 μg/mL for the 7 nm AuNP.

The 18 nm gold particles were synthesized using citrate to reduce Au^3+^ following the procedure of Grabar *et al.* [70]. Briefly, the vigorously stirred aqueous tetrachloroauric acid solution (125 mL, 0.25 mM HAuCl_4_ 3H_2_O) was brought to boiling, followed by rapid addition of 12.5 mL of 40 mM sodium citrate to the vortexed HAuCl_4_ solution, which resulted in a color change from pale yellow to dark wine red. The solution was maintained at boiling temperature for 15 min and then removed from heat. Stirring was continued for another 15 min. After the dispersion had cooled down to room temperature, it was centrifuged at 4000 rpm for 30 min and redispersed in millipore water. The final concentration was 200 μg/mL for the 18 nm AuNP.

The 46 and 80 nm gold particles were synthesized by overgrowth of gold onto the 18 nm citrate-capped AuNP (seed suspension), as described in [71]. For 46 nm AuNP, the growth step was carried out by addition of hydroxylamine-hydrochloride (NH_2_OH∙HCl; 3 mL of 0.2 M; Sigma-Aldrich) and 18 nm citrate-capped AuNP dispersion (15 mL) to a solution of HAuCl_4_ (0.25 mM in 270 mL). For 80 nm AuNP, the first growth step was carried out by the addition of NH_2_OH∙HCl (3 mL of 0.2 M) to a solution of gold HAuCl_4_ (0.25 mM in 270 mL of water) followed by addition of the seed dispersion (18 nm AuNP; 30 mL) resulting in spherical NPs of approximately 40 nm in diameter. A second growth step was performed following the same procedure as the first step with adjusted concentrations: 0.25 mM of HAuCl_4_°3H_2_O, 3 mL of 0.2 M of NH_2_OH∙HCl, and 30 mL of 40 nm AuNP. The resulting AuNP were subsequently functionalized with sodium citrate (1.7 mL of 40 mM sodium citrate) and washed by centrifugation at 4000 rpm for 15 min, and 2500 rpm for 15 min, respectively, and redispersed in millipore water. The final concentrations were 220 μg/mL and 200 μg/mL for the 46 and 80 nm AuNP, respectively.

#### Characterization

UV–Vis spectra of the samples were recorded at 25 °C using a Jasco V-670 spectrophotometer (Jasco Europe S.R.L., Milano, Italy), using 10 mm path length quartz cuvettes. As a first approximation, the particle size was determined directly from UV–Vis spectra using the tabulated theoretical data of uncoated spherical gold nanoparticles in water described in Haiss *et al.* [72]. The size of the different AuNPs was qualitatively confirmed by means of TEM. The surface charge of AuNP samples was measured in suspension of 50 μg/mL in water at 25 °C using a phase amplitude light scattering (PALS) zeta potential analyzer (Brookhaven Instruments Corporation, Hotsville, NY, USA). The Smoluchowski approximation [73] was fitted to 15 cycles of electrophoretic mobility (EPM) measurements and 10 replicates were obtained for each sample to estimate the mean and SD. Dynamic light-scattering (DLS) measurements were carried out at room temperature and at a scattering angle of 90°, using a 3D LS spectrometer (LS instruments AG, Fribourg, Switzerland) equipped with a 21 mW HeNe laser (632.8 nm). Data was collected over 4 min, and five independent correlation functions were measured. The corresponding correlation functions were analyzed using the constrained regularized cumulant method [74].

### Cell cultures

#### A549 monocultures

The A549 cell line [34], a human AT II epithelial cell line, was obtained from the American Tissue Type Culture Collection (LGC Promochem, Molsheim, France). Cells (passage number 8–20) were maintained in RPMI 1640 medium (with 25 mM HEPES; Gibco BRL, Life Technologies, Basel, Switzerland) supplemented with 1 % L-glutamine (Gibco BRL), 1 % penicillin/streptomycin (Gibco BRL) and 10 % foetal calf serum (PAA Laboratories, Lucerna-Chem AG, Lucerne, Switzerland). For experimental cultures, cells were seeded at a density of 0.5 x 10^6^ cells/insert on transparent BD Falcon™ cell culture inserts (surface area of 0.9 cm^2^, pores with 3.0 μm diameter, PET membranes for 12-well plates; BD Biosciences, Basel, Switzerland). Inserts were placed in BD Falcon™ tissue culture plates (12-well plates; BD Biosciences) with 1 mL medium in the upper and 2 mL in the lower chamber. The cells were kept at 37 °C in 5 % CO_2_ humidified atmosphere for 7 days (medium changed after 3–4 days).

#### MLE-12 monocultures

The MLE-12 cell line [33], a mouse AT II epithelial cell line, was obtained from the American Tissue Type Culture Collection. Cells (passage number 7–25) were maintained in advanced Dulbecco’s Modified Eagle Medium: Nutrient mixture F-12 (DMEM/F12) medium (Gibco BRL) supplemented with 1 % HEPES (Gibco BRL), 1 % insulin-transferrin-sodium selenite (Sigma-Aldrich), 1 % L-glutamine, 1 % penicillin/streptomycin, 10 nM hydrocortisone (Sigma-Aldrich) and 10 nM β-estradiol (Sigma-Aldrich). For experimental cultures, cells were seeded at a density of 0.5 x 10^6^ cells/insert on transparent BD Falcon™ cell culture inserts (surface area of 0.9 cm^2^, pores with 3.0 μm diameter, PET membranes for 12-well plates). The cell culture inserts were pretreated with 45 μL Matrigel coating solution, containing 3.36 mg/mL BD Matrigel™ basement membrane matrix growth factor reduced (BD Biosciences) in Dulbecco’s modified Eagle Medium (DMEM) medium (Gibco). Inserts were placed in BD Falcon™ tissue culture plates (12-well plates) with 1 mL medium in the upper and 2 mL in the lower chamber. The cells were kept at 37 °C in 5 % CO_2_ humidified atmosphere for 3.5 days.

#### 16HBE14o- monocultures

The 16HBE14o- cell line [75], a human bronchial epithelial cell line, was kindly provided by Dieter Gruenert (passage number P2.54; University California, San Francisco, CA, USA). Cells (passage number 11) were maintained in minimum essential media (MEM) 1x medium (with Earle’s Salts, 25 mM HEPES and without L-glutamine; Gibco BRL), supplemented with 1 % L-glutamine, 1 % penicillin/streptomycin and 10 % foetal calf serum. For experimental cultures, cells were seeded at a density of 0.5 x 10^6^ cells/insert on transparent BD Falcon cell culture inserts (surface area of 0.9 cm^2^, pores with 3.0 μm diameter, PET membranes for 12-well plates). The cell culture inserts were pretreated with 150 μL fibronectin coating solution, containing 0.1 mg/mL bovine serum albumin (Sigma-Aldrich), 1 % bovine collagen Type I (BD Biosciences) and 1 % human fibronectin (BD Biosciences) in LHC Basal Medium (Sigma-Aldrich). Inserts were placed in BD Falcon™ tissue culture plates (12-well plates) with 1 mL medium in the upper and 2 mL in the lower chamber. The cells were kept at 37 °C in 5 % CO_2_ humidified atmosphere for 7 days (medium changed after 3–4 days).

#### The triple cell co-culture (TCCC) system

A TCCC system with A549 alveolar epithelial cells and human blood monocyte derived macrophages and dendritic cells was used. The TCCC systems were prepared as described before [43, 76]. Briefly, A549 CMLs were cultured as described above. At 7 days, the medium was removed from the upper and lower chamber, the inserts turned upside down and the bottom was abraded carefully with a cell scraper. The inserts were then incubated with 150 μL medium containing 125,000 dendritic cells on the basal side of the CMLs for two hours. Afterwards, the nonadherent cells were removed, the inserts turned around again and placed in BD Falcon™ tissue culture plates (12-well plates). In the lower chamber 2 mL of medium and at the apical side of the CMLs 500 μL medium containing 25,000 macrophages was added. Once again the systems were incubated for two hours to allow the macrophages to attach, before nonadherent cells were washed away. The complete TCCC systems were kept with 2 mL medium in the lower chamber and 1 mL in the upper chamber at 37 °C in 5 % CO_2_ humidified atmosphere for 24 h.

### Air-liquid interface cell exposure system (ALICE)

#### Exposure

The medium in the upper chamber of the CMLs/TCCC systems was removed 24 h prior to exposure in the ALICE (to establish the ALI), the medium in the lower chamber was replaced by 1 mL of fresh medium and the basal side of the membrane was abraded carefully with a cell scraper (only for CMLs). Exposure to NPs in the ALICE was carried out as described elsewhere [12, 32]. Briefly, the ALICE consists of three main components: a droplet generator (nebulizer), an exposure chamber and a flow system with an incubation chamber providing temperature and humidity conditions suitable for cell cultivation. A dense cloud of micron-sized droplets is generated by nebulization of 1 mL AuNP suspension using a vibrating membrane droplet generator (Investigational eFlow, PARI Pharma GmbH, Munich, Germany). The dense cloud of droplets generated by the eFlow nebulizer is transported at a flow rate of 5 L/min into the exposure chamber (20 × 20 × 30 cm) where it gently deposits onto cells cultured at the ALI in standard cell culture plates. Droplet deposition occurs due to single particle sedimentation and an effect known as cloud settling, *i.e.,* the cloud of droplets moves like a bulk object rather than like a collection of individual droplets [32]. The flow rate is chosen so that the cloud is diverted to all sides by the ground plate of the exposure chamber to form an almost symmetric pattern of vortices providing gentle, but sufficient mixing to result in uniform special droplet deposition on the cells. Following the exposure in the chamber (which takes about 15 min), the basal sides of the inserts were immediately washed thoroughly in phosphate-buffered saline solution (PBS; Gibco BRL) and placed in new BD Falcon tissue culture plates (12-well plates), to avoid any cross contamination of AuNP that could have diffused directly into the basolateral medium via the gap between the well and the insert. Afterwards, the cells were kept under ALI conditions in 5 % CO_2_ humidified atmosphere at 37 °C.

To obtain the desired exposure concentration, AuNP suspensions were diluted with millipore water immediately before exposure. For the nebulization of ionic gold an ICP standard solution (Fluka) was used.

#### Sample collection

For each exposure condition (cell line, dose, size and time) the surface liquid, the cell monolayer and the basolateral medium from three different inserts were collected.

After exposure, the surface liquid, the cellular monolayer and the basolateral medium (Fig. 1) were collected at various time points (0, 2, 8, 24, 48 or 72 h post-exposure). First, the basolateral medium was collected. Second, to segregate the surface liquid, the apical side of the CML was rinsed twice with 300 μL PBS. At the end, 600 μL of trypsin-EDTA (0.05 %, with phenol red; Gibco BRL) was placed on the apical side of the CML for approximately 20 min. Afterwards, both sides of the membrane were rinsed thoroughly and the trypsin-EDTA solution containing the detached cells was collected. The obtained samples were stored in 1.5 mL Eppendorf tubes in the freezer until they were further processed to quantify the distribution of gold in the system.

Every 24 h, the basolateral medium was replaced by fresh medium and the old medium was also stored in the freezer until the samples were further processed (*i.e.,* for the 48 and 72 h post-exposure CMLs). The amount of gold in the basolateral medium of each day was summed up to determine the total translocation fraction. A significant influence of the medium replacement on possible saturation effects can be ruled out because the basolateral medium compartment is several times larger than the surface liquid and CML compartment combined and, thus, the concentration of AuNP in the basolateral medium is at all times much lower than in the CML compartment.

#### Surface liquid height

The surface liquid height of the CMLs was quantified by determining the liquid volume with the pipette after 24 h at the ALI. The results represent the mean of the liquid volumes of ten CMLs.

#### The translocated <30 kDa fraction

To assess the stability of the AuNP, the <30 kDa fraction in the basolateral medium from three A549 CMLs 24 h post-exposure was determined. To this end, a centrifugal concentrator with a 30 kDa cutoff filter (Sartorius Vivaspin 2, Sartorius AG, Goettingen, Germany) was used. The samples were centrifuged for 10 min at 500 rpm. The amount of gold in the basolateral solution of the membrane was measured using inductively coupled plasma mass spectrometry (ICP-MS) as described below. The efficiency of the procedure was determined by spiking a 1 mL RPMI medium supplemented as described above with 34 ng gold (gold standard for ICP; Fluka) and comparing the ICP-MS measurements of the spiked samples before and after filtration. As a worst case consideration, the difference in the recovery efficiency before and after filtration was used to correct the translocated <30 kDa fractions.

### Inductively coupled plasma mass spectrometry (ICP-MS)

#### Sample preparation

To determine the total amount of settled AuNP in the ALICE, three wells of the BD Falcon™ tissue culture plates were kept completely empty during exposure. These empty wells were rinsed thoroughly with 1 mL of aqua regia. The aqua regia solutions were then left overnight on the shelf under the fume hood in 15 mL tubes before they were diluted with millipore water to 3 mL for the ICP-MS measurement.

Samples (surface liquid, cellular monolayer and the basolateral medium) were heated up to 70 °C and treated with 500 μL HNO_3_ (Sigma-Aldrich). After two hours at 70 °C, the samples were placed in 15 mL tubes (BD Falcon Conical Tubes, BD Biosciences), supplemented with 500 μL of aqua regia (HCl (Fluka)/HNO_3_ = 1/3, volume ratio) and left overnight on the shelf under the fume hood before they were diluted with millipore water to 3 mL for the ICP-MS measurement. Compared to the settled amount of AuNP in the empty wells, the average recovery rate was approximately 80 % and 75 % for A549 and MLE-12 CMLs (total transwell chamber system), respectively.

#### Measurement

ICP-MS analyses were carried out on an Agilent 8800 ICP-MS (Agilent Technologies, Waldbronn, Germany) using an external calibration curve (1000 μg/ml gold standard for ICP; Merck) with internal standardization (1000 μg/ml iridium standard for ICP; Merck). The instrument is equipped with two quadrupole mass analyzers (MS/MS) and a collision/reaction cell. Rinsing was done with 1 % HNO_3_/2 % HCl between each measurement.

The LOD of the ICP-MS corresponded to a dose of 25 ng/cm^2^. With lower doses it was not possible to reliably determine the translocated AuNP fraction in the basolateral medium 24 h post-exposure.

### Epithelial membrane integrity tests and cell morphology

#### Laser scanning microscopy (LSM)

The CMLs were labelled and scanned as described in earlier publications [13, 77]. Briefly, CMLs were washed in PBS and fixed for 15 min at room temperature in 4 % paraformaldehyde (Sigma-Aldrich) in PBS. Fixed cells were permeabilized in 0.2 % Triton X-100 (Fluka) in PBS for 15 min. The CMLs were incubated with the antibodies at room temperature for two hours. The cytoskeleton (F-actin-filaments) was stained with rhodamine phalloidin 1:50 (R-415; Molecular Probes, Life Technologies Europe B.V., Zug, Switzerland) and the DNA was stained with DAPI 1:100 (Sigma Aldrich). Afterwards, preparations were washed three times in PBS and mounted in Glycergel (Dako Schweiz AG, Baar, Switzerland).

A Zeiss LSM 710 Meta with an inverted Zeiss microscope (Axio Observer.Z1, Lasers: HeNe 633 nm, and Ar 488 nm; Carl Zeiss AG, Feldbach, Switzerland) was used. Image processing and visualization was performed using IMARIS, a three-dimensional multi-channel image processing software for confocal microscopic images (Version 7.4.2; Bitplane AG, Zurich, Switzerland). To visualize the surfaces of the CMLs, a shadow projection was applied.

#### Permeability to Blue Dextran

After 24 h at the ALI, the medium at the basal side of the CML was replaced by 1 mL RPMI medium (without phenol red; Gibco BRL) supplemented with 10 % foetal calf serum. At the apical side of the CML 250 μL of that medium and 250 μL of 1 % Blue Dextran (GE Healthcare; VWR International GmbH, Dietikon, Switzerland) in PBS was placed. For the positive control group, RPMI medium (without phenol red) and 20 mM of EDTA (Sigma-Aldrich) was used. The CMLs were then incubated at 37 °C in 5 % CO_2_ humidified atmosphere for two hours. Afterwards, the basolateral medium was collected and the mean absorbance at 600 nm was measured in triplicate using a Micro-plate Reader (Benchmark Plus; Bio-Rad Laboratories AG, Cressier, Switzerland). In total, the translocation through three CMLs was determined for each cell type. The absorbance was normalized to the translocation of Blue Dextran through an empty insert.

#### Transepithelial electric resistance (TEER) measurements

TEER was measured with the Millicell-ERS system (MERS 000 01; Millipore AG, Volketswil, Switzerland) as described in earlier publications [43, 77]. The mean of three measurements per insert was determined and in total ten inserts per cell line were assessed daily (five inserts without exposure and five inserts with exposure in the ALICE). Before each measurement the CMLs were allowed to equilibrate for 15 min at room temperature in the biological safety cabinet. The electrical resistance of inserts without cells was subtracted from all samples, and the resistance values were multiplied with the surface area of the inserts (0.9 cm^2^).

### Transmission electron microscopy (TEM)

#### Particle diameter before and after ALICE exposure

TEM images were obtained using a Tecnai F20 (FEI, Eindhoven, The Netherlands) equipped with an UltraScan 1000XP 2 k CCD camera (Gatan Inc., Pleasanton, CA, USA). To determine the particle diameters after nebulization in the ALICE system, standard TEM copper mesh grids were placed at the bottom of the ALICE. The images were analyzed automatically using ImageJ for Windows (Version 1.48a; National Institute of Health, Bethesda, MD, USA). By applying the watershed algorithm the individual particles were identified, and then the major diameter and circularity were automatically determined.

#### Intracellular particles

Intracellular particles were visualized by conventional TEM as described before [12]. For TEM analysis, the exposed cells on the transwell membrane were fixed with 2.5 % glutaraldehyde (Sigma-Aldrich) in 0.15 M HEPES buffer for at least 24 h. Then they were washed with HEPES buffer, post-fixed with 1 % osmium tetroxide in sodium cacodylate buffer, washed with maleate buffer, and stained en bloc with 0.5 % uranyl acetate in maleate buffer. Afterwards, the cells were dehydrated in ascending ethanol series, and embedded in Epon. From the embedded cells, ultrathin sections were cut parallel to the vertical axis of the cells and mounted on copper grids, stained with lead citrate and uranyl acetate. TEM images were obtained using a Morgagni 268 (FEI).

### Statistics

An independent groups *t*-test was performed to compare the mean translocation fractions between two CMLs. To this end, the Statistics Calculator for Windows (Version 4.0; StatPac, Inc., Bloomington, MN, USA) was used. The criterion for statistical significance was p ≤ 0.05.

### Physiologically based pharmacokinetic (PBPK) modeling

The PBPK model for AuNP (Fig. 2) was adapted from our recently presented PBPK model for TiO_2_ NPs [39]. The only changes made were the addition of the inhalation/instillation pathway, the harmonization of the investigated organs/compartments with the data of Kreyling *et al.* [20] and the modification of one kinetic rate (*i.e.,* the translocation rate from the organ to the blood). A detailed description of the PBPK model can be found in Additional file 1. In brief, the main idea of the PBPK model is that organs with the same capillary wall type (*i.e.,* the non-sinusoidal non-fenestrated blood capillary type, the non-sinusoidal fenestrated blood capillary type and the sinusoidal blood capillary type, respectively) have the same uptake rate for nanoparticles and that the biodistribution via this transcapillary pathway is size-independent. A sensitivity analysis for the model structure can be found in the Supplemental Information for Bachler *et al.* [39], which reveals that the most sensitive parameters are the blood flow to the respective organs and the translocation constants. For the here-discussed AuNP-model also the translocation rates from the lung deposition compartment will be among the most sensitive parameters.

For all calculations with the PBPK model an uncertainty analysis was conducted using a Monte Carlo simulation (1000 iterations). To this end, log-normal distributions were used for the physiological and compound-dependent parameters, which were changed randomly in each iteration within their given distributions. A full list of parameters, along with the associated equations is given in Additional file 1.

## Additional file

Additional file 1: **Supplementary Material.** Contents: Chapter 1: Additional data on the AuNP characterization: UV–Vis spectra, size distribution histograms, TEM images and physicochemical parameters and dose metrics of the *in vivo* studies that were compared to the CMLs. Chapter 2: Additional data on the CML characterization: LSM images of the CMLs after 72 and 96 h at the ALI and an in-detail discussion on the integrity test with Blue Dextran. Chapter 3: Additional data on the translocation kinetics: Estimation of the number of AuNP per cell exposed and the time-, size- and dose-dependent disposition of the AuNP between the three compartments of the transwell chamber system. Chapter 4: Additional data on the PBPK model: Model methodology, parameterization, parameters, equations and assumptions. And, the predicted translocation fraction and biodistribution of 200 nm gold particles in rats 24 h after intratracheal instillation and the predicted biodistribution of 20 nm AuNP in mice after two hours of inhalation exposure.

## Abbreviations

ALI: Air-liquid interface
ALICE: Air-liquid interface cell exposure
AT II: Alveolar type II
AuNP: Gold nanoparticles
CML(s): Cellular monolayer(s)
DLS: Dynamic light-scattering
EDTA: Ethylenediaminetetraacetic acid
GIT: Gastrointestinal tract
ICP: Inductively coupled plasma
LSM: Laser scanning microscope
LOD: Limit of detection
MS: Mass spectrometry
NP(s): Nanoparticle(s)
PBPK: Physiologically based pharmacokinetic
PBS: Phosphate buffered saline
SD: Standard deviation
TCCC: Triple cell co-culture
TEER: Transepithelial electrical resistance
TEM: Transmission electron microscopy
TiO_2_: Titanium dioxide
