# Supplementary material for: Translocation of gold nanoparticles across the lung epithelial tissue barrier: Combining in vitro and in silico methods to substitute in vivo experiments
Source: Part Fibre Toxicol. 2015 Jun 27;12:18. doi: 10.1186/s12989-015-0090-8 (PMC4483206; doi:10.1186/s12989-015-0090-8)
Supplement: Supplementary file 1 — Supplementary Material. Contents: Chapter 1: Additional data on the AuNP characterization: UV–Vis spectra, size distribution histograms, TEM images and physicochemical parameters and dose metrics of the in vivo studies that were compared to the CMLs. Chapter 2: Additional data on the CML characterization: LSM images of the CMLs after 72 and 96 h at the ALI and an in-detail discussion on the integrity test with Blue Dextran. Chapter 3: Additional data on the translocation kinetics: Estimation of the number of AuNP per cell exposed and the time-, size- and dose-dependent disposition of the AuNP between the three compartments of the transwell chamber system. Chapter 4: Additional data on the PBPK model: Model methodology, parameterization, parameters, equations and assumptions. And, the predicted translocation fraction and biodistribution of 200 nm gold particles in rats 24 h after intratracheal instillation and the predicted biodistribution of 20 nm AuNP in mice after two hours of inhalation exposure. [file 12989_2015_90_MOESM1_ESM.pdf]

Supplementary Material

Translocation of gold nanoparticles across the lung epithelial tissue barrier:

Combining *in vitro* and *in silico* methods to substitute for *in vivo* experiments

Gerald Bachler[1,2], Sabrina Losert[1,3], Yuki Umehara[2], Natalie von Goetz[1,], Laura Rodriguez-Lorenzo[2], Alke Petri-Fink[2], Barbara Rothen-Rutishauser[2], Konrad Hungerbuhler[1]*

[1] ETH Zürich, Institute for Chemical and Bioengineering, 8093 Zürich, Switzerland

[2] University of Fribourg, Adolphe Merkle Institute, 1700 Fribourg, Switzerland

[3] EMPA, Swiss Federal Laboratories for Material Science and Technology, 8600 Dübendorf, Switzerland

*Correspondence: Dr. Natalie von Goetz
ETH Zurich, Institute for Chemical and Bioengineering
Vladimir-Prelog-Weg 1, 8093 Zurich, Switzerland
Tel: +41-44-632-0975
E-mail: natalie.von.goetz@chem.ethz.ch

Contents

1. AuNP Characterization S2

2. CML Characterization S4

3. Translocation Kinetics S6

4. PBPK Modeling S9

4.1. PBPK Model Methodology and Parameterization S9

4.1.1. Absorption S9

4.1.2. Distribution S9

4.1.3. Metabolism S10

4.1.4. Excretion S11

4.1.5. Parameterization S11

4.2. Additional Results S13

4.3. PBPK model Parameters, Equations and Assumptions S16

4.3.1. Physiological Parameters S16

4.3.2. Compound-dependent Parameters S16

4.3.3. Model Assumptions S18

4.3.4. Model Equations S19

5. References S20

1. AuNP Characterization

The most important physicochemical parameters of the gold nanoparticles (AuNP) used are already presented in Table 1 in the main text. Here we additionally present the full UV-Vis spectra (Figure S1) and the size distribution histograms together with TEM images (Figure S2A-E) of the AuNP.

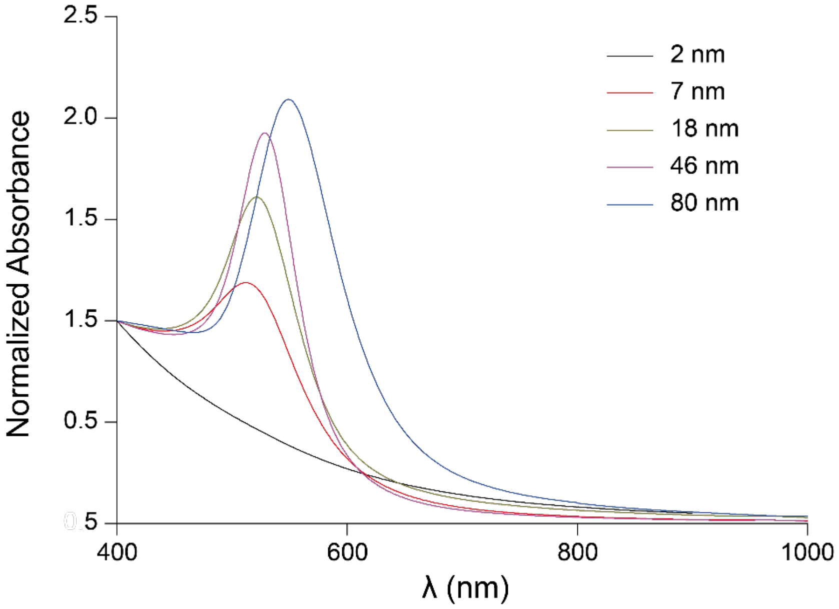

Figure S1: UV-Vis spectra of the different sizes of the AuNP.

In Table S1 the physicochemical parameters and dose metrics are listed for the *in vivo* studies that were compared to the cellular monolayers (CMLs).

Table S1: Physicochemical parameters and dose metrics of AuNP that were used by Kreyling *et al.* [1] and Schleh *et al.* [2]

Source	Diameter (nm)	Dose[a] (ng/cm^2)	Surface functionalization[c]	ζ-potential (mV)
	1.4	0.65 ± 0.10	S-TPP (SO_3^-)	-20.6 ± 0.5
	2.8[b]	0.20 ± 0.03	TGA (COO^-)	negative
**Kreyling *et al.* [1]**	5	8.63 ± 0.50	S-TPP (SO_3^-)	-21.1 ± 1.4
	18	0.63 ± 0.05	S-TPP (SO_3^-)	-22.8 ± 3.1
	80	4.40 ± 0.15	S-TPP (SO_3^-)	-23.3 ± 1.6
**Schleh *et al.* [2]**	20	3.31	n.a.[d]	n.s. (-40)[e]

[a] mean ± standard deviation (SD); Kreyling *et al.* [1]: based on a lung surface area of 4000 cm^2; Schleh *et al.* [2]: based on a particle weight of 8 x 10^{-17} g (only deposited particles were considered).

[b] Kreyling *et al.* used negatively and positively (CA (NH_3^+); dose: 4.43±0.32 ng/cm^2) charged 2.8 nm large AuNP. Detailed information was only provided by Kreyling *et al.* [1] for the negatively charged particles. However, the biodistribution of both particle types normalized to the amount of translocated gold is practically identical.

[c] S-TPP (SO_3^-) – sulfonated triphenylphosphine; TGA (COO^-) – thioglycolic acid.

[d] not applicable; Particles were produced with a spark ignition generator.

[e] not specified by Schleh *et al.* [2]; However, Masereel *et al.* [3] reported that uncoated AuNP may have a zeta potential of approximately -40 mV.

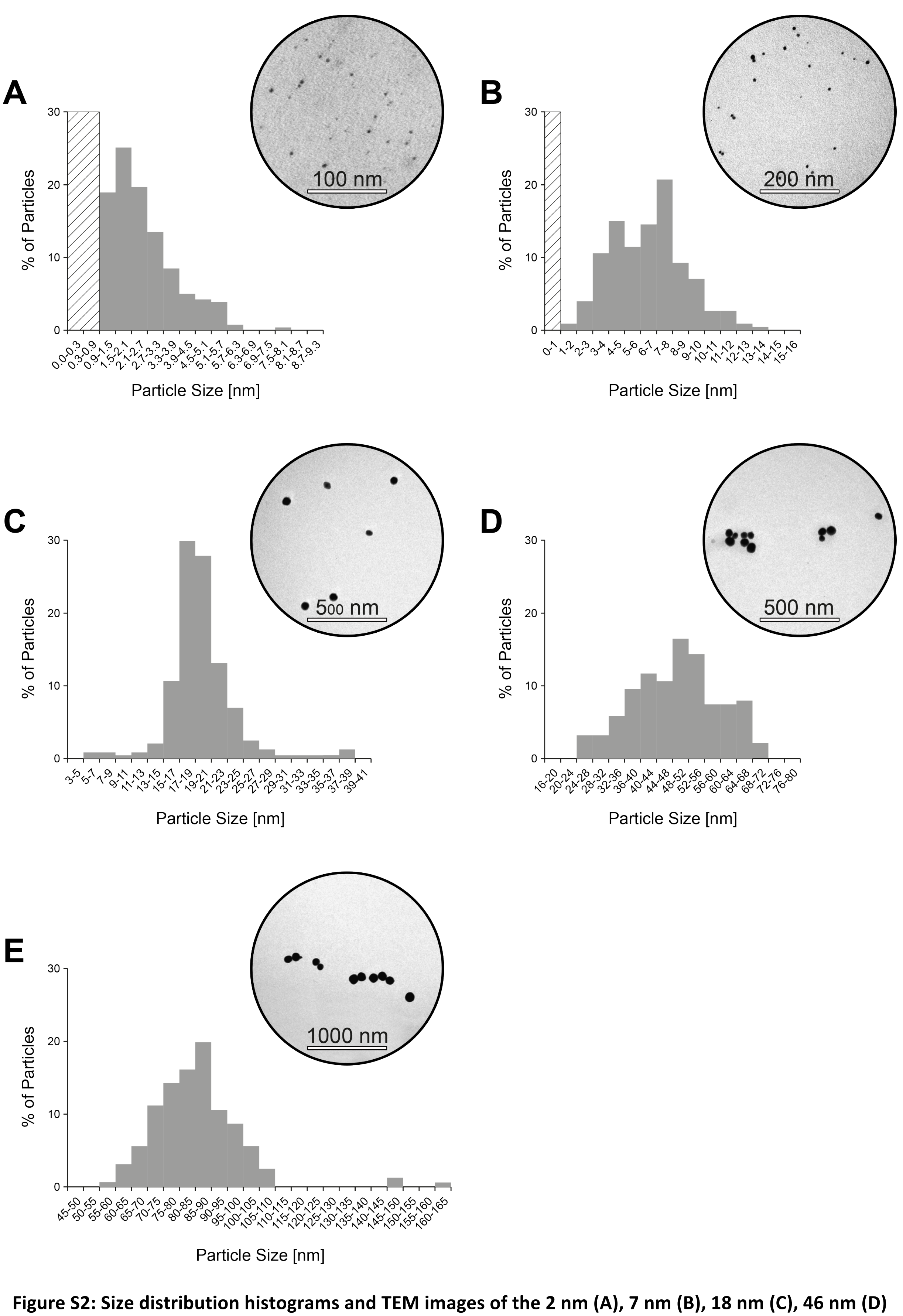

Figure S2: Size distribution histograms and TEM images of the 2 nm (A), 7 nm (B), 18 nm (C), 46 nm (D) and 80 nm (E) large gold particles after ALICE exposure.
Number of analyzed particles: 259 (2 nm), 227 (7 nm), 244 (18 nm), 188 (46 nm) and 161 (80 nm).
(shaded areas represent the limit of detection).

2. CML Characterization

The laser scanning microscope (LSM) images of the CMLs after 24 and 48 hours at the air-liquid interface (ALI) are already illustrated in the main part of the paper. Here we additionally show the images of the CMLs after 72 and 96 hours at the ALI (Figure S3A-D), which corresponds to 48 and 72 hours after exposure in the air-liquid interface cell exposure (ALICE) system.

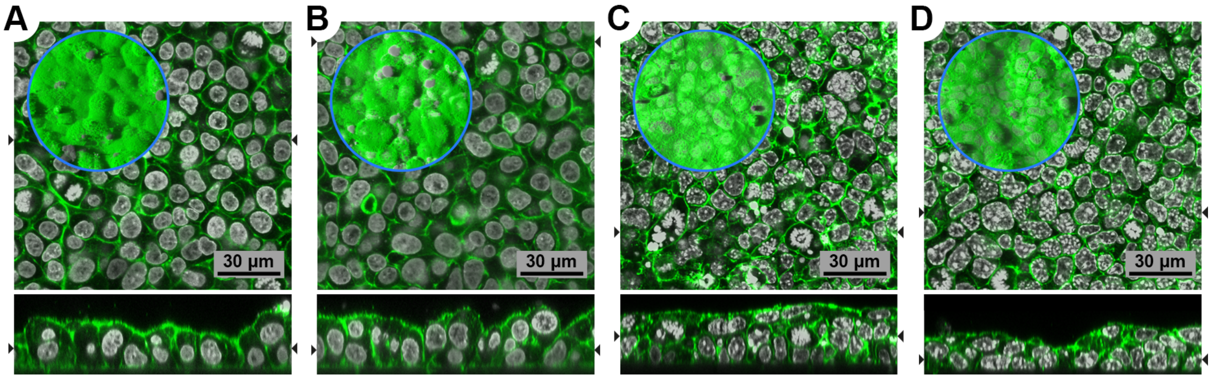

Figure S3: LSM images of cytoskeletal F-actin (green) and the cell nuclei (grey). Orthogonal view of the A549 CML (A) 72 and (B) 96 hours, and of the MLE-12 CML (C) 72 and (D) 96 hours at the ALI. After 24 hours at the ALI, the CMLs were exposed to 100 ng/cm^2 18 nm AuNP. Within the blue circle the top view on the CML is depicted.

The integrity of the CMLs was tested by assessing the permeability to dextran, an approach that is commonly used to evaluate the permeability of epithelial barriers [4-7]. We exposed the CMLs to Blue Dextran and measured the translocation within two hours from the apical side to the basolateral medium (Figure S4). A relative absorbance of 1 corresponds to a Blue Dextran translocation that is equal to the translocation through an empty insert. As reference control, 16HBE14o- human bronchial epithelial CMLs were used, which are known to form a tight barrier [8]. As positive control, the CMLs were additionally exposed to ethylenediaminetetraacetic acid (EDTA), which causes the detachment of the CML from the membrane [9] and, hence increases the translocation of Blue Dextran. As can be seen, the A549 CML was as tight as the 16HBE14o- CML at all time points. The MLE-12 CML was slightly more permeably than the reference control. However, a significantly increased translocation of Blue Dextran through the A549/MLE-12 CMLs compared to the 16HBE14o- CML was observed only for the MLE-12 CML after 48 hours at the ALI ($p < 0.01$). The addition of EDTA caused a significantly increased translocation of Blue Dextran from the apical side of the CML to the basolateral medium for both cell types and at all time points ($p < 0.01$; except MLE-12 CMLs after 96 hours at the ALI: $p < 0.05$). The increased translocation of Blue Dextran after the addition of EDTA was less pronounced for the MLE-12 CMLs. This is probably due to the fact that the membrane was additionally coated with Matrigel, which supports the attachment of the cells.

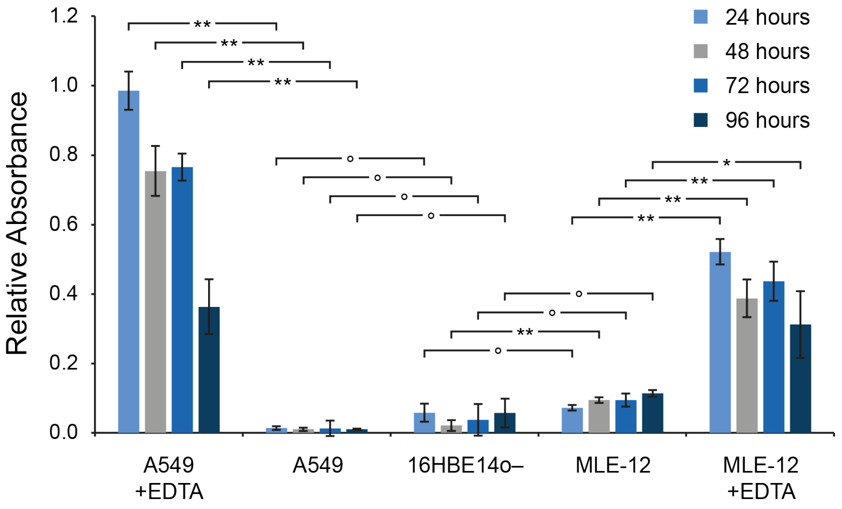

Figure S4: Integrity of A549 and MLE-12 CMLs at various time points at the ALI. The translocation of Blue Dextran (2,000 kDa) through the CMLs was compared to 16HBE14o- CMLs (reference control) and CMLs additionally exposed to EDTA (positive control). (data are expressed as mean±SD, n=3 CMLs; ⌐¬ indicates the significance level between two measurements ° no significant difference, significant difference: * p < 0.05, ** p < 0.01).

Transepithelial electrical resistance (TEER) measurements were carried out to evaluate the integrity of the CMLs (Figure S5). Both cell types show a very similar TEER time course, with a slightly lower resistance for MLE-12 CMLs. In general, the TEER of the CMLs increased especially during the first 24 hours after seeding and at the ALI, respectively. A steady rise in the TEER can also be observed from day 9 onwards. This rise is associated with the formation of a second cellular layer (Figure S3), which develops when the CMLs are longer than 48 hours at the ALI. Exposure to AuNP in the ALICE did not significantly change the TEER of A549 and MLE-12 CMLs 24, 48 and 72 hours post-exposure.

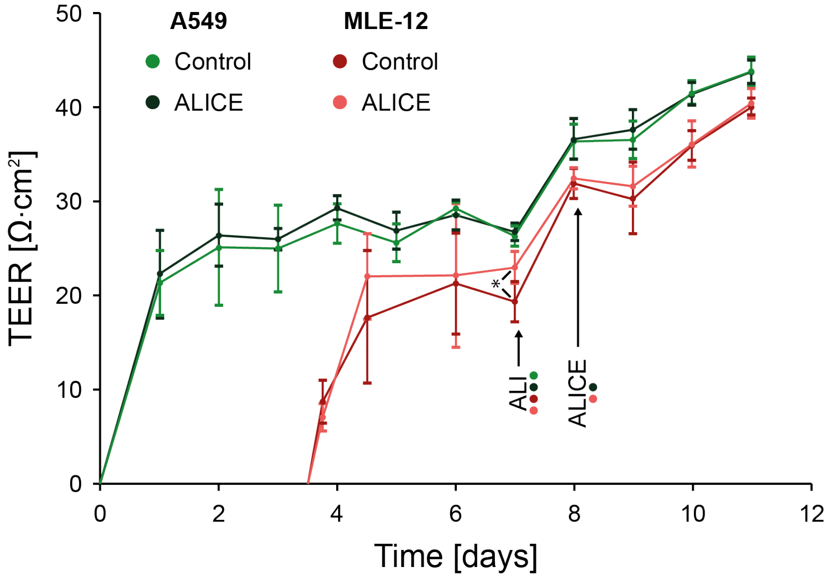

Figure S5: The TEER of the A549 and MLE-12 CMLs over time and the influence of exposure to 100 ng/cm² of 18 nm large AuNP at day 8. (data are expressed as mean±SD, n=5 CMLs; significant difference: * p < 0.05).

3. Translocation Kinetics

Based on the number of cells/cm^2 (obtained from the LSM images) we estimated roughly the number of nanoparticles (NPs) per cell that settle on the CMLs in the ALICE system. The number of AuNP per cell for the different particle sizes and doses are summarized in Table S2.

Table S2: Estimation of the number of NPs per cell that settle on the CMLs in the ALICE system

AuNP Size (nm)	Dose (ng/cm^2)	# NPs/cell	
		**A549 CML**	**MLE-12 CML**
**2**	100	2,700,000	2,100,000
**7**	100	62,000	49,000
**18**	25	1’100	850
	50	2,200	1,700
	100	4,400	3,400
	150	6,500	n.a.[a]
	200	8,700	6,800
**46**	100	220	170
**80**	100	42	33

[a] not applicable

In Figure S6, the gold distribution between the surface liquid, the cell monolayer and the basolateral medium is shown for both cell types (A549 and MLE-12) and all incubation time periods (Figure S6A), particle sizes (Figure S6B) and doses (Figure S6C). The translocation of AuNP to the basolateral medium and the time-dependent disposition is already extensively discussed in the main part of the paper. The results for the size-dependent disposition (Figure S6B) also show a larger fraction of gold in the surface liquid of MLE-12 CMLs compared to A549 CMLs, which is most likely linked to a higher surface liquid height in MLE-12 CMLs. With decreasing particle size this difference becomes less pronounced, which can be attributed to the fact that smaller particles can pass easier through the CML. The dose-dependent disposition (Figure S6C) is very much the same for all doses in both particle types, with the exception of the decreased translocation in A549 CMLs at a dose larger than 100 ng/cm^2. Also here, one can see the larger fraction of gold in the surface liquid of MLE-12 CMLs as comparted to A549 CMLs.

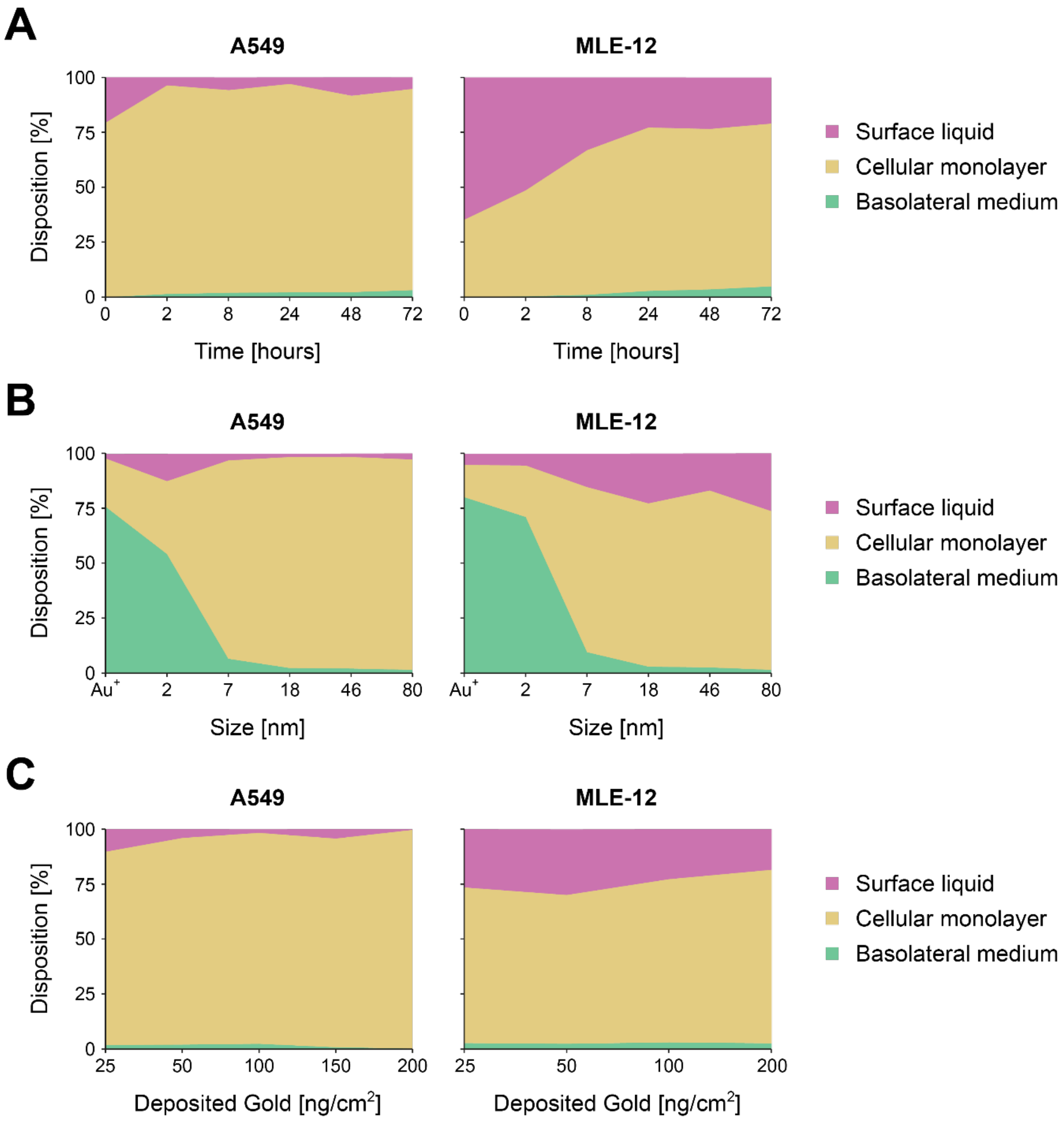

Figure S6: Disposition of AuNP and ionic gold in the transwell chamber system for A549 and MLE-12 CMLs. (A) 18 nm AuNP at an exposure dose of 100 ng/cm^2 varying with time. (B) An exposure dose of 100 ng/cm^2 AuNP after 24 hours for various particle sizes. (C) 18 nm AuNP after 24 hours for various doses. The results of the translocated fraction (green areas) are presented in more detail in Figure 5.

In Figure S7A-E, transmission electron microscopy (TEM) images of the A549 CML 24 hours after exposure to 2, 7 or 46 nm large AuNP are depicted. As already discussed in the main text, after exposure of the CML to 2 nm AuNP we could observe only one large agglomerate (ca. 300 nm), which is shown in Figure S7B. Single particles or even small agglomerates did not have enough contrast to be distinguished from the background. However, this indicates that the 2 nm AuNP were also mainly present as single particles or very small agglomerates. The disposition of the 7 and 46 nm AuNP within the CML was very similar to the 18 nm large particles (Figure 8). The AuNP were mainly present as single particles or as small agglomerates and freely distributed within the cytoplasm (Figure S7B-D) and next to the cell nucleus (Figure S7E). Particles in vesicles were not observed.

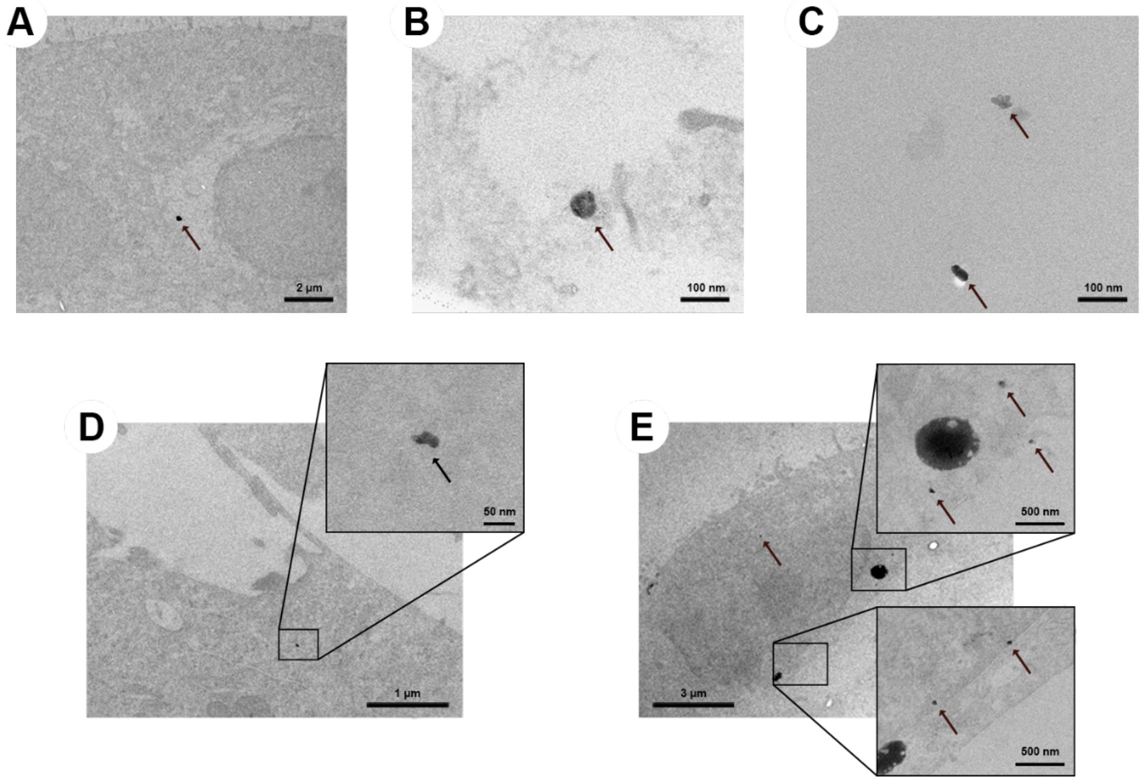

Figure S7: TEM images of intracellular particles in the A549 CML. Agglomerates of (A) 2 nm and (B,C,D) 7 nm AuNP in the cytoplasm and (E) 46 nm AuNP in and next to the cell nucleus. 24 hours after exposure to 100 ng/cm^2 AuNP in the ALICE system. The arrows are pointing towards the particles. (all images without lead citrate and uranyl acetate staining).

4. PBPK Modeling

4.1. PBPK Model Methodology and Parameterization

Our recently presented physiologically based pharmacokinetic (PBPK) model on titanium dioxide (TiO_2) NPs [10] was used for the simulation of the absorption, distribution, metabolism and excretion (ADME) of AuNP. A detailed discussion of the model methodology can be found in Bachler *et al.* [10]. Here we will give just a brief overview on the model concept and the modifications that were implemented for AuNP.

4.1.1. Absorption

In the AuNP PBPK model, only the absorption of AuNP after inhalation and instillation are considered (no dermal or intestinal uptake). To this end, two sub-compartments were added to the lung compartment of the PBPK model: the lung deposition compartment and the lung deposition storage compartment (Figure 2). The inhaled/instilled AuNP are added to the lung deposition compartment. From this compartment, the particles are then either translocated to the blood or to the lung deposition storage compartment. Since the *in vivo* data that was used to determine the pulmonary absorption is only available for the first 24 hours after exposure [1], it is currently not possible to model the long-term behavior (*e.g.* permanent storage, mucociliary clearance, translocation to the blood circulation) of the AuNP in the lung deposition storage compartment. Hence, at the moment the lung deposition storage compartment is modeled as a sink for AuNP. For the simulations that are presented in this work this uncertainty about the long-term behavior is of minor importance, because the maximum simulated time is 72 hours. However, as indicated by our *in vitro* experiments, in addition to the very fast translocation within the first couple of hours after exposure, there might be a slow translocation of preliminarily stored AuNP from lung cells to the blood circulation, which becomes significant after 2-3 days. Hence, for long-term simulations with the PBPK model such kinetics have to be considered and possibly added to the model.

4.1.2. Distribution

The biodistribution of TiO_2 NPs was modeled according to:

- their ability to cross the capillary wall of the organs
- the phagocytosis capability by the mononuclear phagocyte system (MPS)

However, for the distribution of NPs to the MPS compartments (*i.e.* in the liver, lung and spleen) we determined a threshold concentration of NPs in the blood that is necessary to trigger the uptake of NPs by these compartments [10, 11]. In the *in vivo* studies that are presented in this work [1, 2] the concentrations of NPs in the blood are below this threshold. Hence, the MPS compartments were

excluded in the AuNP PBPK model. Furthermore, in the TiO_2 NPs PBPK model, compartments were implemented for the skin, intestines, stomach and bones. These organs/tissues were not individually evaluated by Kreyling *et al.* [1], but were part of the “Remainder” compartment. Consequently, to be in accordance with the *in vivo* data, these four compartments were merged with the remainder compartment of the PBPK model.

The translocation rate from the blood to the organs was determined for the transcapillary pathway, with the following equation:

$$k_{trans\_blood\_organ} = \mathrm{b}_{\mathrm{trans\_constant\_organ}} * \frac{Q_{organ\_blood}}{V_{blood}} \quad \text{(S1)}$$

where $b_{trans\_constant\_organ}$ [-] represents the translocation constants for NPs which depend on the capillary wall type (CT) of the organ and were taken from the TiO_2 NPs PBPK model, Q_{organ_blood} [L/min] is the flux of blood through the organ and V_{blood} [L] is the total blood volume in the body. The CTs and the corresponding $b_{trans\_constant\_organ}$ values can be found in Table S4 and Table S5, respectively.

In the TiO_2 NPs PBPK model, also compartments are present that describe the deposition and temporary storage of TiO_2 NPs in the different organs. However, since these kinetics only play a role in simulations that are longer than one week, these compartments were also left out in the AuNP PBPK model (maximum simulation time is three days). However, for long-term simulations with the PBPK model such kinetics have to be considered and possibly added to the model.

4.1.3. Metabolism

Like TiO_2 NPs, AuNP are very stable and do not dissolve under *in vivo* conditions [1, 12]. Hence, dissolution kinetics were not implemented.

Furthermore, as any nanoparticle that comes into contact with a biological medium, AuNP are immediately binding proteins to their surfaces [13]. This protein corona guides the excretion and biodistribution of NPs [14]. For silver NPs, we could already show with the PBPK model that the surface of the particles is only of minor importance for the simulation of the biodistribution [11]. This finding is further supported by the study of Kreyling *et al.* [1], who could show that for 2.8 nm AuNP with a positive (NH_3^+) or negative (COO^-) zeta potential, an identical biodistribution was observed. Solely the gold concentrations in the kidneys are significantly different (student’s t test: $p < 0.05$). However, the particles have a diameter that is close to the cut-off size for the renal excretion of NPs (ca. 5 nm) [15] and, hence, slight differences in the size distribution can strongly influence the uptake of NPs by the kidneys and the renal excretion of NPs. Thus, it was assumed that the formed protein

corona is comparable between the different types of AuNP and consequently surface-dependent kinetics were also not considered (as it was also done in the TiO_2 NPs PBPK model).

4.1.4. Excretion

The same biliary and urinary excretion rates as in the TiO_2 NPs PBPK model were used.

4.1.5. Parameterization

As discussed above, the structure and parameters of the AuNP PBPK model were taken largely from our recent publication on TiO_2 NPs [10]. The assumptions and parameters for the AuNP PBPK model are summarized in Table S3-S7. Hence, for the AuNP PBPK model only three parameters had to be determined: the translocation rate from the organs to the blood, the translocation rate of the deposited AuNP to the blood and the storage rate of the deposited AuNP in the lung. To this end, the *in vivo* data after instillation of 18 nm large AuNP from Kreyling *et al.* [1] was used.

The translocation rate from the organs to the blood was increased compared to the TiO_2 NPs PBPK model to consider the lower retention time of AuNP in the organs as compared to TiO_2 NP. This was done by increasing the kinetic rate manually until the comparison of the *in vivo* data of Kreyling *et al.* [1] to the results of the PBPK model showed a good conformity (Figure S8).

The lung storage and translocation rate (for the 18 nm particle) were fitted so that the lung gold levels (80±14% dose/g organ) and the translocation fraction one hour after exposure (0.32±0.083%, Figure S9) matched the *in vivo* data, respectively. It is important to mention that Kreyling *et al.* [1] did not determine the biliary excretion of AuNP, due to the inability to distinguish biliary excreted AuNP from AuNP cleared via the mucociliary route. Hence, the amount of translocated AuNP is decreasing with time in the data of Kreyling *et al.* (Figure S9). With the PBPK model it is, however, possible to distinguish AuNP excreted via the biliary route from AuNP cleared via the mucociliary route. Thus, the *in vivo* data was adjusted with the PBPK model for the biliary excretion of AuNP, so that the total translocation of AuNP through the air-blood barrier for the data of Kreyling *et al.* could be determined (Figure S9).

The lung translocation rates for the other particle sizes (*i.e.* 2, 7, 46 and 80 nm) were fitted with the adjusted translocation fractions 24 hours post-exposure from Kreyling *et al.* [1] (Figure 5B). The adjustment of the translocation fractions 24 hours post-exposure was carried out by increasing the translocation fractions with a factor of 3.9, which was determined for 18 nm AuNP as described above.

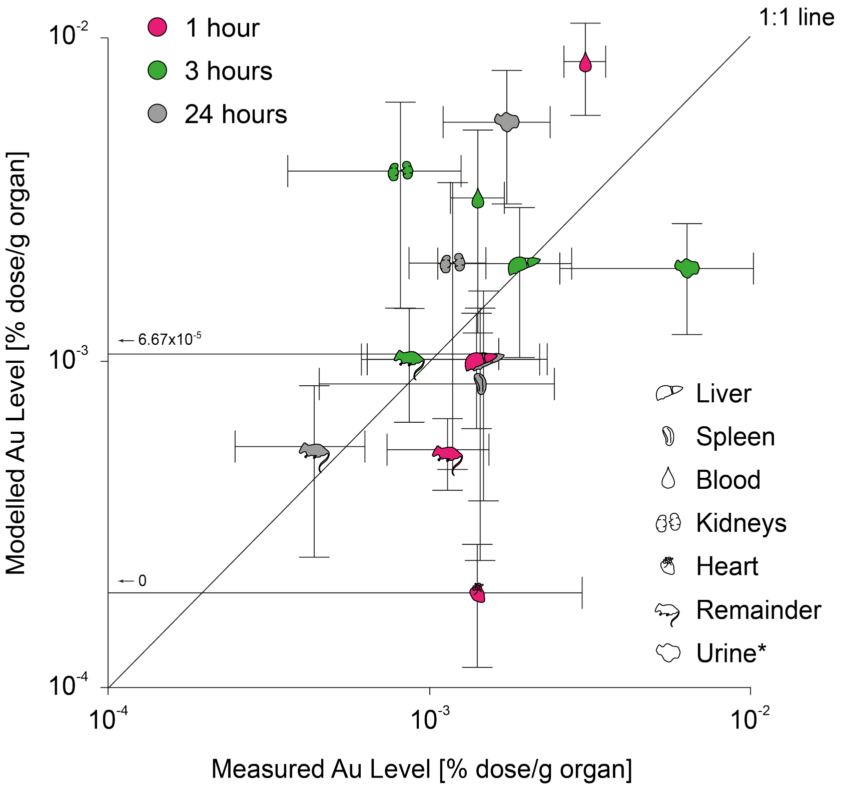

Figure S8: Results of the PBPK model calibration with the data of Kreyling *et al.* [1] at different time points after instillation of 18 nm AuNP in the rat lung (*in % dose; data are expressed as mean±SD, n=4 (rats) and n=1000 iterations (PBPK model)).

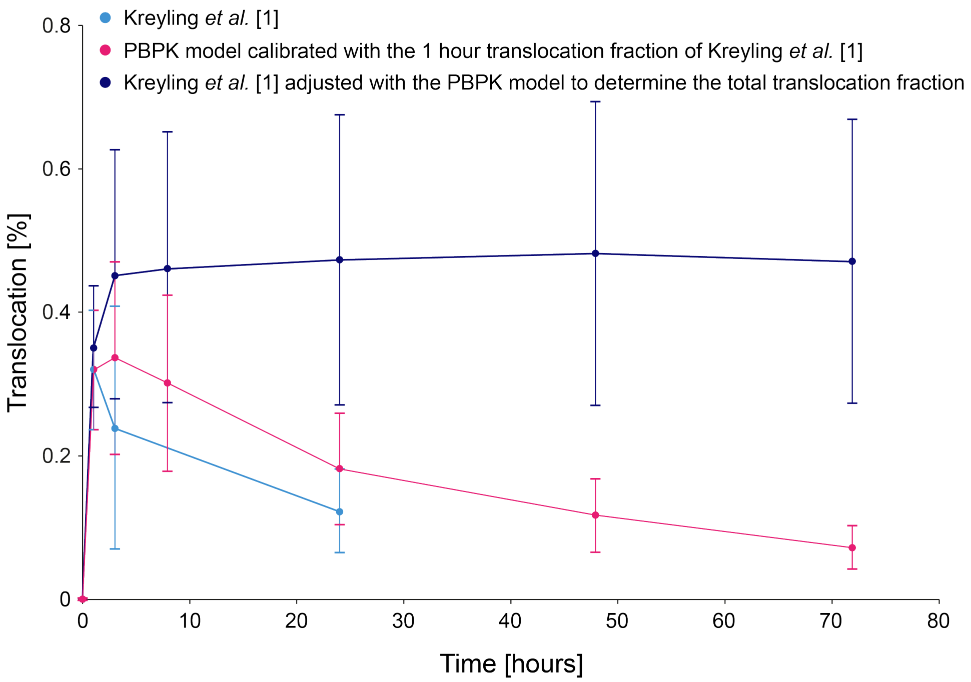

Figure S9: Comparison of the translocation fractions through the air-blood barrier of Kreyling *et al.* [1] (light blue) and the PBPK model (18 nm AuNP instillation in rat lung, dark blue). With the PBPK model it was further possible to consider the biliary excretion of AuNP and, hence, to determine the total translocation of AuNP across the air-blood barrier (red, data are expressed as mean±SD, n=4 (rats) and n=1000 iterations (PBPK model)).

4.2. Additional Results

In Figure S10, the comparison of the gold levels in the lung between the measured levels by Kreyling *et al.* [1] and the predicted levels by the PBPK model are depicted.

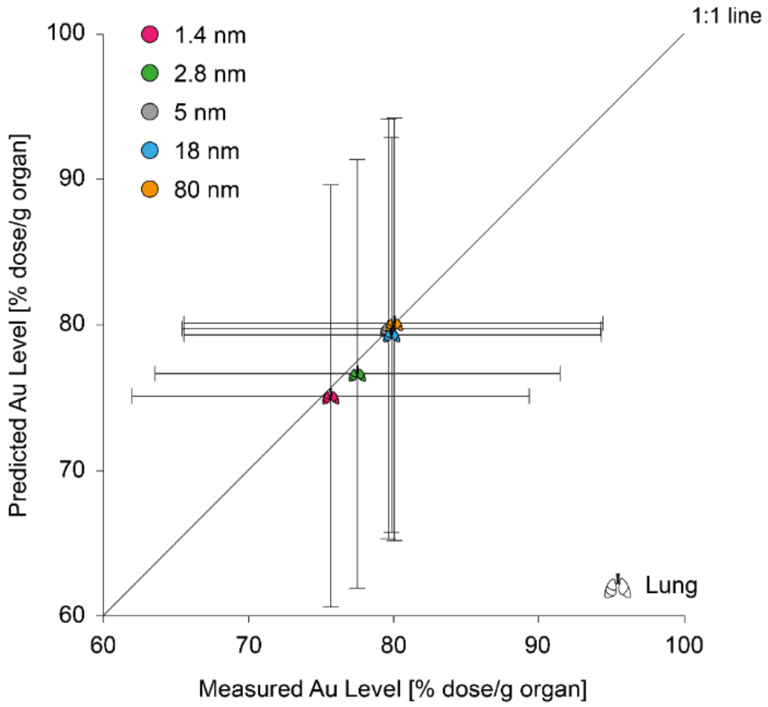

Figure S10: Comparison of the PBPK model to biokinetic data of female Wistar-Kyoto rats 24 hours after intratracheal instillation of various sizes of AuNP [1] (data are expressed as mean±SD, n=4 (rats) and n=1000 iterations (PBPK model)).

The size-dependent translocation kinetics of gold particles through the air-blood barrier of Wistar-Kyoto rats are shown in Figure S11. The curves presented are the same as in Figure 5B, but complemented by the translocation fraction for 200 nm gold particles that was reported by Kreyling *et al.* [1]. The adjusted translocation fraction for 200 nm, however, was determined with a decreased translocation rate from the blood to the liver and a decreased biliary excretion rate, because the PBPK model is only applicable for particles up to 150 nm [11], based on the pore size of the liver capillaries [16]. For particles above this size limit it may be more difficult to pass the pores of the capillary wall of the liver and it can be expected that larger particles are not that easily transported to the liver tissue and are hardly excreted via the biliary pathway. This is basically the same threshold phenomenon that can be seen in the kidneys for AuNP that are larger than 5 nm. Once the particles are larger than the pore size of the kidney capillaries, they are transported at a much lower rate to the kidneys and urine (Figure 9). Thus, for 200 nm large gold particles the same liver translocation rate and biliary excretion rate was chosen as for the kidneys and urinary excretion, respectively (assuming that 200 nm particles are now larger as the capillary pore size of the liver). Based on this assumption, the translation rate from the lung to the blood for 200 nm gold particles was fitted with

the *in vivo* data from Kreyling *et al.* [1] using the same approach as described above for the 18 nm AuNP (*i.e.* fitting the lung translocation rate so that the translocation fraction 1 hour post-exposure is 0.16%, which was measured in rats). Unfortunately, there is only indirect evidence for a decreased biliary excretion of particles above 150 nm at the moment (*e.g.* [17] or the fact that the reported translocation fractions by Kreyling *et al.* [1] are decreasing over time for all particle sizes, but not for 200 nm large gold particles, where the translocation fraction is in fact increasing with time). However, the adjusted translocation fraction for 200 nm gold particles fits much better in the course of the curve as compared to the one reported by Kreyling *et al.* [1] (Figure S11), indicating that the assumptions on the size threshold are plausible.

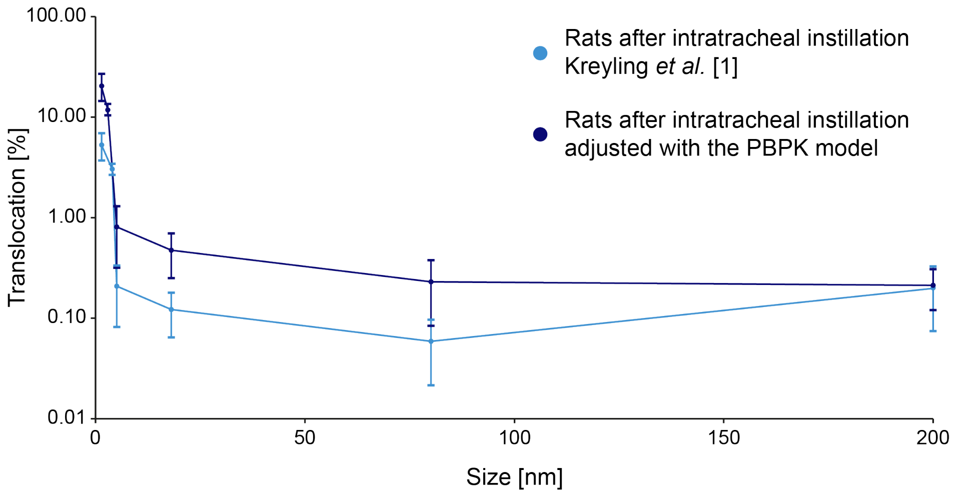

Figure S11: Size-dependent translocation kinetics of various sizes of gold particles 24 hours after intratracheal instillation. *In vivo* data from female Wistar-Kyoto rats was taken from Kreyling *et al.* [1] and corrected for biliary excretion with the PBPK model (for 200 nm a decreased biliary excretion was assumed) (data are expressed as mean±SD, n=4 (rats) and n=1000 iterations (PBPK model)).

The assumptions of a decreased biliary excretion and translocation to the liver of 200 nm gold particles is further supported by the modeled biodistribution with the modified PBPK model. As can be seen in Figure S12, the biodistribution can be well predicted when the assumptions are considered in the PBPK model. Except for the kidneys' gold level, the gold levels of all organs fall close to the 1:1 line. However, it should be noted that the PBPK model was not developed and validated for such large particles. Other factors, which are not considered in the PBPK model, may also influence the biodistribution of particles above 150 nm. For example, Rejman *et al.* [18] reported that particles below 200 nm are predominantly internalized via clathrin-mediated pathways by non-phagocytic B 16 cells, while particles larger than 200 nm are to a higher rate internalized via caveolae-mediated pathways. Nevertheless, it seems that the PBPK model can, to a certain degree, also be used for particles above 150 nm, but it should be kept in mind that the predictive capability of the PBPK model above this size limit has not been properly evaluated yet.

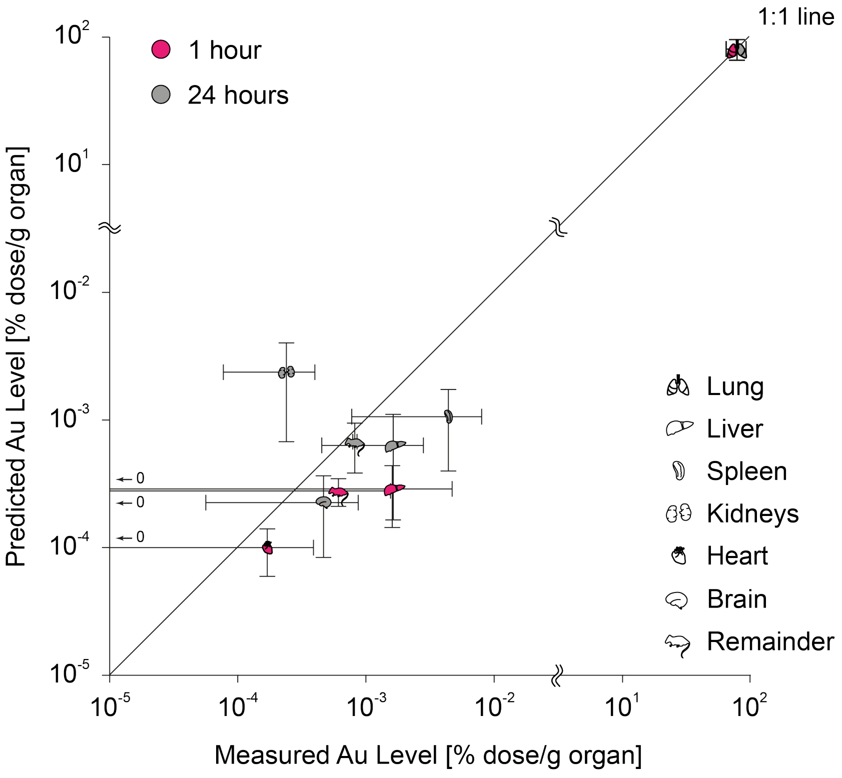

Figure S12: Comparison of the PBPK model to biokinetic data of female Wistar-Kyoto rats of different time points after intratracheal instillation of 200 nm large gold particles [1] (only organs where the gold levels in all rats were above the LOD are depicted; data are expressed as mean±SD, n=4 (rats) and n=1000 iterations (PBPK model)).

In Figure S13, the biodistribution of AuNP in mice was predicted by using the exposure conditions of Schleh *et al.* [2] as input parameters for our PBPK model.

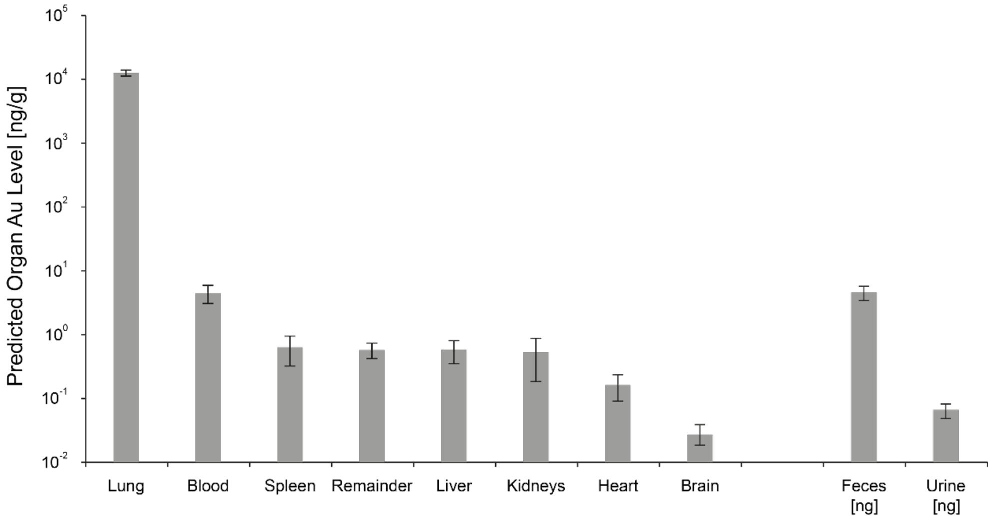

Figure S13: Biodistribution of 20 nm AuNP in mice immediately after two hours of inhalation exposure (total deposition 3.31 ng/cm^2) as predicted by the PBPK model. The translocation fraction (1.2%, adjusted for biliary excretion: 1.42%) was fitted to match the *in vivo* data from Schleh *et al.* [2] (data are expressed as mean±SD, n=1000 iterations).

4.3. PBPK model Parameters, Equations and Assumptions

4.3.1. Physiological Parameters

The physiological parameters for mice and rats are summarized in Table S3. For the Monte Carlo simulation the blood flow to the organs was normalized to the average organ weight.

Table S3: Physiological parameters for weight and blood flow (mean±SD) of organs in % of total body weight and % of cardiac output, respectively. If not indicated otherwise, parameter values were taken from Brown *et al.* [19]

Organ	Mouse		Rat	
	Weight [%]	Blood Flow [%]	Weight [%]	Blood Flow [%]
Liver	5.49 ± 1.32	16.10 ± 0.20	3.66 ± 0.65	18.30 ± 2.50
Kidneys	1.67 ± 0.17	9.10 ± 2.90	0.73 ± 0.11	14.10 ± 1.90
Spleen	0.35 ± 0.16	1.13 ± 0.34[a,b]	0.20 ± 0.05	0.85 ± 0.26[a,b]
Heart	0.50 ± 0.07	6.60 ± 0.90	0.33 ± 0.04	5.10 ± 0.10
Brain	1.65 ± 0.26	3.30 ± 0.30	0.57 ± 0.14	2.00 ± 0.30
Lung	0.73 ± 0.08	1.92 ± 0.87[c]	0.50 ± 0.09	2.10 ± 0.40
Remainder	89.61 ± 1.37	75.96 ± 3.20	94.01 ± 0.68	72.85 ± 3.19
Cardiac Output [L/min]	0.0140 ± 0.0029		0.110 ± 0.016	
Blood [kg]	0.0018 ± 0.0001[d]		0.016 ± 0.001[e]	
Body Weight [kg]	0.02[a]		0.250[a]	

[a] Davies and Morris (1993)
[b] No data for the standard deviation is available. Hence, the standard deviation was estimated based on the standard deviation from the other organs.
[c] Stott et al. (1983)
[d] Riches et al. (1973)
[e] Lee and Blaufof (1985)

4.3.2. Compound-dependent Parameters

The organs were grouped according to their CT and in total, four CT groups were identified (adapted from Sarin [16], see also Table S4):

- CT1: non-sinusoidal non-fenestrated blood capillary type (*i.e.* brain, heart, lung),
- CT2: non-sinusoidal fenestrated blood capillary type (*i.e.* kidneys),
- CT3: sinusoidal blood capillary type with pores larger than 15 nm (*i.e.* liver, spleen),
- CT4: the "Remainder" compartment, which represents all tissues and organs that are not covered by any other compartment, hence, could not be assigned to a specific CT group.

Table S4: Classification of blood capillary type and the physiological upper limit of pore size according to Sarin [16] as used for defining the capillary wall type groups (The CT4 describes the permeability of the "Remainder" compartment and, hence covers various capillary types)

Acronym	Capillary type	Sub-Type	Physiological upper limit of pore size	Representative tissue	Model Compartments
CT1	Non-sinusoidal non-fenestrated type	with tight junctions	<1nm	Brain, Spinal Cord, Lymphoid Tissue Cortex	Brain, Lung, Heart
		with loose junctions	~5nm	Skin, Muscles, Lung, Cortical Bone, Intestinal Mesentery	
CT2	Non-sinusoidal fenestrated type	diaphragmed fenestrae	6-12nm	Skin, Testis, Kidney Peritubular, Intestinal Mucosa	Kidneys
		open fenestrae	~15nm	Kidney Glomerulus	
CT3	Sinusoidal reticuloendothelial type	open fenestrae	~180nm<br>~280nm[a]	Liver	Liver
CT3	Sinusoidal non-reticuloendothelial type	terminal capillary ending	~5µm	Splenic red pulp arterial blood capillary	Spleen
		interendothelial slits	—	Splenic red pulp venous blood capillary	

[a] Mouse and rat

The compound-dependent parameters that describe the distribution and excretion, and absorption of AuNP are summarized in Table S5 and Table S6, respectively. The listed translocation constants for NPs (CT1-4, biliary and urinary excretion) were directly taken from our recently presented PBPK model for TiO_2 NPs [10]. The translocation rate from the organs to the blood circulation, the pulmonary absorption rate and the lung storage rate were fitted to match the *in vivo* data of the 18 nm AuNP from Kreyling *et al.* [1]. The pulmonary absorption rates of the other particle sizes were fitted with the according *in vivo* data (Kreyling *et al.*: 1.4, 2.8, 5, 80 and 200 nm; Schleh *et al.*: 20 nm), which is discussed in more detail in the main text. For more information on the model methodology and model parameterization see the discussion above ("PBPK Model Methodology and Parameterization").

Table S5: Compound-dependent parameters for the distribution and excretion of AuNP (mean±SD).

	Acronym	Value [10^{-4}]	Unit
**Nanoparticulate translocation constants[a]**	$b_{trans\_constant\_organ}$		
CT1		0.622 ± 0.194	-
CT2[b]		4.97 ± 2.31	-
CT3		9.95 ± 2.74	-
CT4		34.2 ± 4.09	-
Biliary excretion		61.3 ± 3.28	-
Urinary excretion[b]		1.55 ± 0.0830	-
**Translocation rate from the organ to the blood[c]**	$k_{trans\_organ\_blood}$	33.0 ± 11.3	min^{-1}

[a] Which was used for the calculation of the translocation rate [min^{-1}] of NPs from the blood to the organs/excrements - see Equation S1

[b] For the 200 nm particles the CT2 translocation constant and urinary excretion constant were used to determine the translocation rate from the blood to the liver and feces, respictively. This was done according to the idea of the PBPK model that partiles of this size cannot pass the capillary wall of the liver as easy as smaller particles due to the pore size of the capillary wall.

[c] Decreased by a factor of 10 for the brain compartment to consider the blood-brain barrier

Table S6: Compound-dependent parameters for the absorption of AuNP (mean±SD).

		Acronym	Value [10^{-4}]	Unit
**Translocation rate from the lung deposition to the blood**		$k_{trans\_lung\_blood}$		
**Kreyling *et al.* [1]**	1.4 nm		76.7 ± 7.38	min^{-1}
	2.8 nm		36.4 ± 3.50	min^{-1}
	5 nm		2.29 ± 0.220	min^{-1}
	18 nm		1.30 ± 0.125	min^{-1}
	80 nm		0.631 ± 0.0606	min^{-1}
	200 nm		0.568 ± 0.0546	min^{-1}
**Schleh *et al.* [2]**	20 nm		5.93 ± 0.570	min^{-1}
**Storage rate of the deposited gold in the lung**		$k_{storage\_lung}$	300 ± 120	min^{-1}

4.3.3. Model Assumptions

Table S7: Summary of the assumptions made for the AuNP PBPK model

**General**	• The mixing of nanoparticles in all compartments is instantaneous and complete.<br>• First-order kinetics is describing reasonably well the processes *in vivo*.
**Distribution**	• Blood flow is non-pulsatile and constant.<br>• Distribution of nanoparticles in the blood stream is size-independent and the particles do not alter the blood flow rate.<br>• Endocytosis processes are size-independent (which does not hold for the absorption of AuNP to the blood circulation in the lung).<br>• The organ uptake of AuNP via the capillary wall is proportional to the amount of gold that passes the capillary walls.<br>• For AuNP the transport efficiency through the capillary wall is similar between mice and rats.

4.3.4. Model Equations

Table S8: Model equations of the AuNP PBPK model (for mice and rats). The model parameters and rates used are listed in Table S3-S6 (“A” denotes the amount of gold in the corresponding compartment)

**Translocation rate from the blood to the organs[a]:**		$k_{trans\_blood\_organ} = \mathrm{b}_{\mathrm{trans\_constant\_organ}} * \frac{Q_{organ\_blood}}{V_{blood}}$
**Absorption[b]**	Deposition in the lung	$\frac{\mathrm{d}A_{deposition\_lung}}{\mathrm{d}t} = \dot{m}^{c}_{deposition} - k_{storage\_lung} * A_{deposition\_lung} - \dot{m}_{absorption}$
	Absorption to the blood	$\dot{m}_{absorption} = k_{trans\_lung\_blood} * A_{deposition\_lung}$
	(Temporary) Storage in the lung[d]	$\frac{\mathrm{d}A_{deposition\_storage\_lung}}{\mathrm{d}t} = k_{storage\_lung} * A_{deposition\_lung}$
**Distribution**	Blood compartment:	$\frac{\mathrm{d}A_{blood}}{\mathrm{d}t} = \dot{m}_{absorption} + \sum_{Organs^{e}} (\mathrm{k}_{\mathrm{trans\_organ\_blood}} * A_{organ}) - \sum_{Organs^{a}} (k_{trans\_blood\_organ} * A_{blood})$
	Organ[e] compartment:	$\frac{\mathrm{d}A_{organ}}{\mathrm{d}t} = k_{trans\_blood\_organ} * A_{blood} - \mathrm{k}_{\mathrm{trans\_organ\_blood}} * A_{organ}$
**Excretion**	Biliary excretion:	$\frac{\mathrm{d}A_{biliary\_excretion}}{\mathrm{d}t} = k_{trans\_blood\_organ-biliary} * A_{blood}$
	Urinary excretion:	$\frac{\mathrm{d}A_{urinary\_excretion}}{\mathrm{d}t} = k_{trans\_blood\_organ-urinary} * A_{blood}$

[a] Organ: brain, excrements (biliary and urinary excretion), heart, kidneys, liver, lung, remainder and spleen compartment

[b] Absorption: Inhalation and instillation

[c] $\dot{m}_{depsosition}$: for Kreyling *et al.* [1] – instillation: deposition of all gold within one minute; for Schleh *et al.* [2] – inhalation: deposition 13.5 ng/min for two hours (in total 1.7 µg or 3.31 ng/cm^2)

[d] The long-term behavior of this “stored” gold is unknown. The available *in vivo* data from Kreyling *et al.* [1] is only available for the first 24 hours after exposure (see also main text)

[e] Organ: brain, heart, kidneys, liver, lung, remainder and spleen compartment

5. References

1. Kreyling WG, Hirn S, Moller W, Schleh C, Wenk A, Celik G, Lipka J, Schaffler M, Haberl N, Johnston BD, et al: **Air-blood barrier translocation of tracheally instilled gold nanoparticles inversely depends on particle size.** *ACS Nano* 2014, **8:**222-233.
2. Schleh C, Holzwarth U, Hirn S, Wenk A, Simonelli F, Schaffler M, Moller W, Gibson N, Kreyling WG: **Biodistribution of inhaled gold nanoparticles in mice and the influence of surfactant protein D.** *J Aerosol Med Pulm Drug Deliv* 2013, **26:**24-30.
3. Masereel B, Dinguizli M, Bouzin C, Moniotte N, Feron O, Gallez B, Borght TV, Michiels C, Lucas S: **Antibody immobilization on gold nanoparticles coated layer-by-layer with polyelectrolytes.** *J Nanopart Res* 2011, **13:**1573-1580.
4. Kelly JJ, Moore TM, Babal P, Diwan AH, Stevens T, Thompson WJ: **Pulmonary microvascular and macrovascular endothelial cells: differential regulation of Ca^{2+} and permeability.** *Am J Physiol-Lung C* 1998, **274:**L810-L819.
5. Bouwmeester H, Poortman J, Peters RJ, Wijma E, Kramer E, Makama S, Puspitaninganindita K, Marvin HJ, Peijnenburg AA, Hendriksen PJ: **Characterization of translocation of silver nanoparticles and effects on whole-genome gene expression using an in vitro intestinal epithelium coculture model.** *ACS Nano* 2011, **5:**4091-4103.
6. Elbert KJ, Schafer UF, Schafers HJ, Kim KJ, Lee VHL, Lehr CM: **Monolayers of human alveolar epithelial cells in primary culture for pulmonary absorption and transport studies.** *Pharm Res* 1999, **16:**601-608.
7. Salomon JJ, Muchitsch VE, Gausterer JC, Schwagerus E, Huwer H, Daum N, Lehr CM, Ehrhardt C: **The Cell Line NCl-H441 Is a Useful in Vitro Model for Transport Studies of Human Distal Lung Epithelial Barrier.** *Mol Pharm* 2014, **11:**995-1006.
8. Sakagami M: **In vivo, in vitro and ex vivo models to assess pulmonary absorption and disposition of inhaled therapeutics for systemic delivery.** *Adv Drug Delivery Rev* 2006, **58:**1030-1060.
9. Mahida YR, Makh S, Hyde S, Gray T, Borriello SP: **Effect of Clostridium difficile toxin A on human intestinal epithelial cells: Induction of interleukin 8 production and apoptosis after cell detachment.** *Gut* 1996, **38:**337-347.
10. Bachler G, von Goetz N, Hungerbuhler K: **Using physiologically based pharmacokinetic (PBPK) modeling for dietary risk assessment of titanium dioxide (TiO_2) nanoparticles.** *Nanotoxicology* 2014, **early online**.
11. Bachler G, von Goetz N, Hungerbuhler K: **A physiologically based pharmacokinetic model for ionic silver and silver nanoparticles.** *Int J Nanomedicine* 2013, **8:**3365-3382.
12. Schmidt J, Vogelsberger W: **Dissolution kinetics of titanium dioxide nanoparticles: the observation of an unusual kinetic size effect.** *J Phys Chem B* 2006, **110:**3955-3963.
13. Landsiedel R, Fabian E, Ma-Hock L, Wohlleben W, Wiench K, Oesch F, van Ravenzwaay B: **Toxico-/biokinetics of nanomaterials.** *Arch Toxicol* 2012, **86:**1021-1060.
14. Goppert TM, Muller RH: **Polysorbate-stabilized solid lipid nanoparticles as colloidal carriers for intravenous targeting of drugs to the brain: Comparison of plasma protein adsorption patterns.** *J Drug Target* 2005, **13:**179-187.
15. Choi HS, Liu W, Misra P, Tanaka E, Zimmer JP, Itty Ipe B, Bawendi MG, Frangioni JV: **Renal clearance of quantum dots.** *Nat Biotechnol* 2007, **25:**1165-1170.
16. Sarin H: **Physiologic upper limits of pore size of different blood capillary types and another perspective on the dual pore theory of microvascular permeability.** *J Angiogenes Res* 2010, **2:**1-14.
17. Hirn S, Semmler-Behnke M, Schleh C, Wenk A, Lipka J, Schaffler M, Takenaka S, Moller W, Schmid G, Simon U, Kreyling WG: **Particle size-dependent and surface charge-dependent biodistribution of gold nanoparticles after intravenous administration.** *Eur J Pharm Biopharm* 2011, **77:**407-416.
18. Rejman J, Oberle V, Zuhorn IS, Hoekstra D: **Size-dependent internalization of particles via the pathways of clathrin- and caveolae-mediated endocytosis.** *Biochem J* 2004, **377:**159-169.
19. Brown RP, Delp MD, Lindstedt SL, Rhomberg LR, Beliles RP: **Physiological parameter values for physiologically based pharmacokinetic models.** *Toxicol Ind Health* 1997, **13:**407-484.
20. Davies B, Morris T: **Physiological parameters in laboratory animals and humans.** *Pharm Res* 1993, **10:**1093-1095.
21. Riches AC, Sharp JG, Thomas DB, Smith SV: **Blood volume determination in the mouse.** *J Physiol* 1973, **228:**279-284.
22. Lee HB, Blaufox MD: **Blood volume in the rat.** *J Nucl Med* 1985, **26:**72-76.

23. Stott WT, Dryzga MD, Ramsey JC: **Blood-flow distribution in the mouse.** *J Appl Toxicol* 1983, **3:**310-312.
